# Systematic profiling of growth interactions in human gut microbiome species

**DOI:** 10.1038/s41467-026-76526-z

**Published:** 2026-08-07

**Authors:** Bolor Buyanbadrakh, Elisabeth Baland, Paula Lambeck, Lucía Pérez Jiménez, Sandra M. Holmberg, Fabiola Puértolas-Balint, Eric Toh, Sun Nyunt Wai, Bjoern O. Schroeder, Madeleine Ramstedt, Shaochun Zhu, André Mateus

**Affiliations:** 1 Department of Chemistry, Umeå University, Umeå, Sweden; 2 Department of Molecular Biology, Umeå University, Umeå, Sweden; 3 The Laboratory for Molecular Infection Medicine Sweden (MIMS), Umeå, Sweden; 4 Umeå Center for Microbial Research (UCMR), Umeå, Sweden; 5 Science for Life Laboratory (SciLifeLab), Umeå, Sweden

**Keywords:** Microbiome, Proteomics, Bacterial systems biology

## Abstract

Microbial interactions shape the composition and stability of the human gut microbiome. Yet, few studies have systematically investigated species-species growth interactions and the mechanisms behind these. Here we show that among 36 representative gut bacterial strains, when two species interact, the interactions are mostly inhibitory. To provide biological insight into specific interactions, we further investigate the basis of a positive interaction, showing that *Clostridium perfringens* promotes the growth of *Mediterraneibacter gnavus* through extracellular vesicles. Additionally, we identify *Veillonella parvula* as a species capable of modulating environmental pH, thereby enabling the growth of *Parabacteroides merdae*, a strain highly sensitive to acidic conditions. This pH-increasing effect is enhanced by guanine supplementation and persists in multi-species communities containing different pH-lowering strains from diverse bacterial phyla. Although *V. parvula* is commonly present in human gut microbiomes, it is generally found at low levels. Given the spatial organization of bacteria in the gut, the local pH modulation by *V. parvula* might support the growth of acid-sensitive strains. Overall, the comprehensive dataset and mechanistic insights presented here provide a starting point to predict microbiome composition by integrating growth interactions.

## Abstract (web summary)

Here, Buyanbadrakh *et al*. systematically map growth interactions among gut bacteria, revealing predominantly inhibitory effects, and identifying extracellular vesicles and pH modulation as mechanisms that enable growth-promoting interactions.

## Introduction

Microorganisms commonly live in complex communities, whose composition is shaped by molecular interactions among the microorganisms that compose them^1,2^. Species may compete for nutrients, secrete inhibitory molecules either into the environment or directly into neighboring cells, or alter the chemical environment (for example, by lowering the pH) to restrict the growth of other species. On the other hand, microorganisms can secrete metabolic by-products that promote the growth of other species. The combined effect of all the interactions results in a community that has a stable composition, unless challenged by strong perturbations such as antibiotic treatment or dietary changes^3–5^. In the context of the human microbiome, this community resilience is critical to prevent colonization by pathogenic species^6–8^.

Studying species interactions is thus key to predict community composition, and ultimately its molecular functions. This can be accomplished by growing one species on sterilized medium in which another species was previously grown (spent medium)^9–11^, or co-culturing pairs of species together and deconvoluting their individual growth, e.g., by measuring the abundance of species-specific DNA sequences^12–15^ or proteins^16^, or by physically separating species while allowing molecular exchange^17^. These pairwise interactions are generally able to predict bacterial behavior in a community^12,14^, but higher-order interactions can also be studied directly by assembling communities of different complexity^18–20^. Alternatively, it is also possible to use computational approaches, such as models based on the metabolic capacity encoded on the genome of each species^21^.

In this work, we use a spent medium approach to systematically profile pairwise growth interactions of 36 representative bacterial strains of the human gut microbiome (Supplementary Data 1). These species cover a large phylogenetic diversity (spanning six major phyla) and exhibit varied abundance and prevalence across individuals^22,23^. Generally, we observe that the majority of interactions are inhibitory and mostly driven by media acidification. To provide insight into specific interactions, we study the *Mediterraneibacter gnavus* growth promotion by *Clostridium perfringens*, which we show is driven by extracellular vesicles. We also highlight how *Veillonella parvula* can increase extracellular pH, and how this enables *Parabacteroides merdae* growth, including in a community setting. These results can be integrated to predict community composition and identify mechanisms to modulate it.

## Results

### Binary growth interactions among human gut microbes

**Fig. 1:**
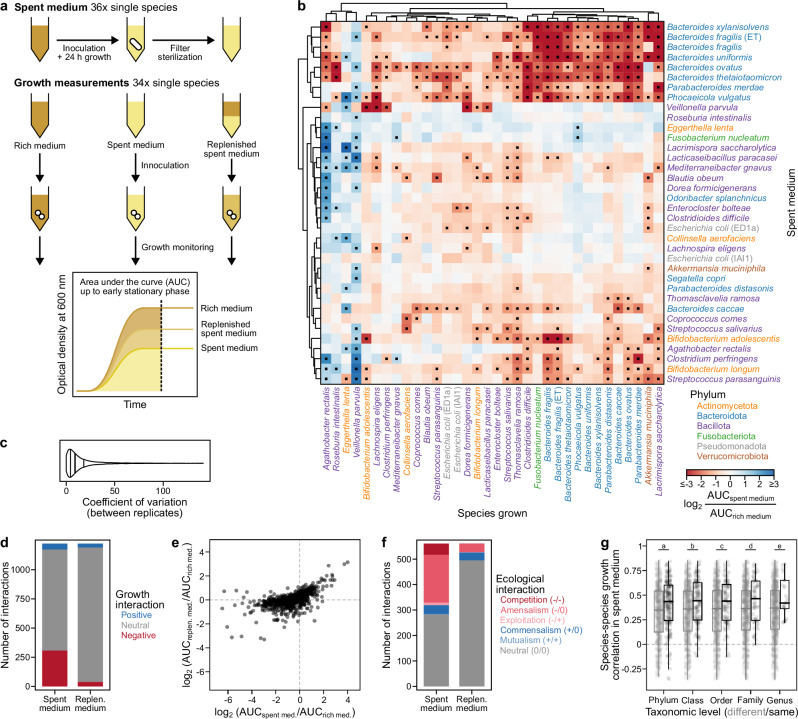
Growth interaction profiling among human gut microbes. **a** Interactions were measured by spent medium experiments in which one of 36 bacterial strains was grown for 24 h in rich medium, followed by filter sterilization. A second strain was then grown in this spent medium, replenished medium, or rich medium as a control and growth was followed by measurements of optical density at 600 nm (OD_600_) of each individual culture. The area under the curve of the early stationary phase (AUC) for each of the conditions was used as the final growth measurement (the order of the schematic growth curves is used for illustration purposes only). **b** Pairwise changes in AUC of strains grown (column) in spent medium (row) of another strain compared to rich medium. Strains are color-coded according to the phylum to which they belong. Cases in which the absolute fold change is > 2 are marked in black.** c** Reproducibility of growth measurements as measured by the coefficient of variation of the AUC across replicates. Median is highlighted with a thicker line and quartiles with thinner lines. **d** Number of interactions with a change of at least 2-fold in spent medium or replenished medium compared to rich medium. **e** Comparison of spent medium and replenished medium experiments. **f** Number of interactions between two strains using an ecological classification to identify reciprocity. **g** Distribution of Pearson correlation coefficients between the growth behavior of pairs of strains (column-wise correlation of values in panel **b**) if they belong or not to the same taxonomic level. Individual data points are shown and box represents the interquartile range, line represents the median, and whiskers represent the 5^th^ and 95^th^ percentiles. Two-sided *t* test: ^a^*t*(403) = 4.2, *p* = 3.9 × 10^−5^, 95% CI [0.049, 0.13]; ^b^*t*(227) = 4.43, *p* = 1.5 × 10^−5^, 95% CI [0.059, 0.15]; ^c^*t*(157) = 3.7, *p* = 2.9 × 10^−4^, 95% CI [0.045, 0.15]; ^d^*t*(92) = 3.7, *p* = 3.3 × 10^−4^, 95% CI [0.052, 0.17]; ^e^*t*(28) = 3.27, *p* = 0.0029, 95% CI [0.049, 0.21].

To study directional growth interactions among human gut bacteria, we used a spent media assay. First, we collected the spent medium of 36 bacterial strains grown for 24 h in a rich medium (Gifu anaerobic medium; GAM). Then, each individual strain was cultured in each of these spent media or nutrient-replenished spent media (spent media mixed with an equal volume of 2x concentrated rich medium). As a control, we grew the same strains in rich medium (Fig. 1a). Growth was monitored by continuously measuring optical density at 600 nm (OD_600_) for 48 h. Growth dynamics were quantified by calculating the area under the growth curve (AUC; Supplementary Data 2). Due to inconsistent day-to-day variation in lag phase, *Segatella copri* and *Odoribacter splachnicus* were excluded from further growth analyses. However, we still assessed how other strains grew on their spent media, because the yield after 24 h was consistent. In total, this resulted in 1224 binary interactions being tested (Fig. 1b and Supplementary Data 2). Growth measurements were highly reproducible, with a median coefficient of variation of 8.5% (interquartile range: 13.6%) for the AUC across replicates (Fig. 1c).

When considering a two-fold change compared to rich medium ( | log_2_ fold change | > 1), we observed 307 inhibitory interactions (25.1%), and 51 interactions where growth was promoted (4.2%) (Fig. 1d). The number of inhibitory interactions decreased by more than 8-fold to 38 interactions (3.1%) when spent medium was replenished with nutrients, with most inhibitory effects becoming neutral (Fig. 1e and Supplementary Fig. 1). This suggests that most inhibitory interactions are due to depleted resources or changes to the media that limit growth (e.g., pH; see below). In contrast, a large proportion of growth promotion interactions (27 out of 58 interactions) were unchanged even after nutrient replenishment, suggesting potential cross-feeding.

We next explored if inhibitory interactions were mostly reciprocal, classified as competition (-/-) in ecology, or if one strain showed exploitation (-/+) or amensalism (-/0). Our results showed that most interactions pointed to amensalism (33.5% of all pairwise interactions), followed by competition (7.8%), with exploitation accounting only for 1.8% of the interactions (Fig. 1f). Only a small fraction of commensalism (+/0; 6.3%) and mutualism (+/+;0.36%) were observed. It should be noted that these relationships are inferred, since strains were not co-cultured. However, they reflect known interactions, for example, we observed that the spent media of *Fusobacterium nucleatum*, *Streptococcus salivarius*, and *S. parasanguinis* promoted the growth of *Veillonella parvula* (log₂ fold-change of 1.4-2.7 compared to rich medium; Supplementary Data 2), which aligns with previous reports showing that *Veillonella* frequently co-occurs with *F. nucleatum* and *Streptococcus* species in oral microbiomes and exhibits strong intergeneric coaggregation^24,25^.

Finally, we noticed that strains that were phylogenetically more closely related tended to behave similarly across the different spent media, as indicated by a stronger correlation in their responses to spent media compared to pairs without a taxonomic match (Fig. 1g), with the effect being stronger the more taxonomically related the two strains were. This indicates that taxonomic similarity may be a contributing factor to how species interact with, and respond to, the metabolic environment shaped by others.

In summary, we generated a comprehensive dataset of pairwise strain interactions across human gut microbiome bacteria. This highlighted a high prevalence of competition due to resource depletion or changes to the ecosystem, but also some cooperative interactions that are important in shaping community composition.

### Clostridium perfringens vesicles enhance the growth of Mediterraneibacter gnavus

**Fig. 2:**
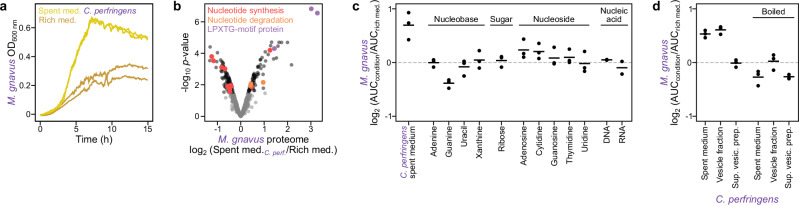
Mechanism of growth enhancement of *Mediterraneibacter gnavus* by *Clostridium perfringens* spent medium. **a** Growth over time as measured by OD_600_ of *M. gnavus* in rich medium or *C. perfringens* spent medium. **b** Proteome abundance changes of *M. gnavus* grown in *C. perfringens* spent medium compared to rich medium. Differential protein abundance was analyzed using limma empirical Bayes moderated two-sided *t* test, with Benjamini-Hochberg false discovery rate (FDR) correction for multiple comparisons. **c** Effect of nucleobases, sugar, nucleosides, and nucleic acids on the growth of *M. gnavus* compared to rich medium. Data from individual biological replicates (triplicates, except for nucleic acid, which shows duplicates) and the median are shown. **d** Effect of vesicle fraction present in *C. perfringens* spent medium, or the supernatant of the purification, on the growth of *M. gnavus* compared to rich medium. All conditions were also boiled prior to measurement. Data from individual biological triplicates and the median are shown.

*Mediterraneibacter gnavus* (formerly *Ruminococcus gnavus*) exhibited enhanced growth when cultured in *Clostridium perfringens* spent medium compared to standard rich medium (Fig. 2a). To investigate the basis of this effect, we performed a quantitative proteomic analysis of *M. gnavus* grown in *C. perfringens* spent medium versus rich medium. The proteomic profile revealed a decreased abundance of proteins involved in de novo nucleotide synthesis, and a concomitant increase in proteins associated with nucleotide metabolism and degradation pathways (Fig. 2b and Supplementary Data 3). This suggested that *M. gnavus* relies less on the synthesis of nucleotides when grown in *C. perfringens* spent medium. Thus, we tested whether nucleic acid components could promote the growth of *M. gnavus* by culturing it in media supplemented with nucleobases, nucleosides, the sugar component (ribose), and nucleic acids. However, none of these supplements showed a growth promotion effect (guanine showed a slight inhibitory effect; Fig. 2c and Supplementary Data 4), suggesting that a more complex or alternative uptake mechanism may be involved.

Previous studies have reported that *C. perfringens* secretes membrane vesicles containing proteins, peptidoglycan, and nucleic acids^26–28^. The proteome analysis of *M. gnavus* also revealed a strong increase in the abundance of three LPXTG-motif cell wall anchor domain proteins (Fig. 2b). Despite having an uncharacterized function, these proteins are generally associated with interactions with the environment (e.g., adhesion or colonization)^29,30^. Using dynamic light scattering, we observed particles with an average diameter of 71 nm in *C. perfringens* spent medium, while rich medium contained particles with an average diameter of 149 nm (Supplementary Fig. 2a). We then isolated the crude extracellular vesicle fraction from the spent medium of *C. perfringens* by ultracentrifugation, and confirmed the presence of particles with an average size of 58 nm (Supplementary Fig. 2a). These particles were visibly different from particles isolated from rich medium, with a hollow core reminiscent of vesicles (Supplementary Fig. 2b). *M. gnavus* exhibited increased growth by 1.4-fold when cultured in rich medium supplemented with vesicles isolated from *C. perfringens* (*t*(2) = 13.5, *p* = 0.0054, 95% CI log_2_ fold change [0.36, 0.7] on a two-sided one-sample *t* test; Fig. 2d and Supplementary Data 5). Importantly, growth was not promoted when *M. gnavus* was grown in the supernatant collected after ultracentrifugation (*t*(2) = -0.29, *p* = 0.79, 95% CI log_2_ fold change [-0.18, 0.16] on a two-sided one-sample *t* test; Fig. 2d), which lacked detectable particles when analyzed by dynamic light scattering (Supplementary Fig. 2a). Furthermore, heat treatment of the vesicle fraction abolished the growth-promoting effect (*t*(2) = 0.27, *p* = 0.80, 95% CI log_2_ fold change [− 0.34, 0.39] on a two-sided one-sample *t* test; Fig. 2d), and led to a change in the morphology of the vesicles (Supplementary Fig. 2b). This indicated that heat either disrupted vesicle structure or that the growth-promoting components are heat-labile.

In summary, *C. perfringens* promotes the growth of *M. gnavus* by releasing extracellular vesicles, which might facilitate the uptake of growth enhancing components, such as nucleic acid components present in these vesicles^26–28^.

### pH changes in the medium are crucial mediators of inter-microbial interactions

**Fig. 3:**
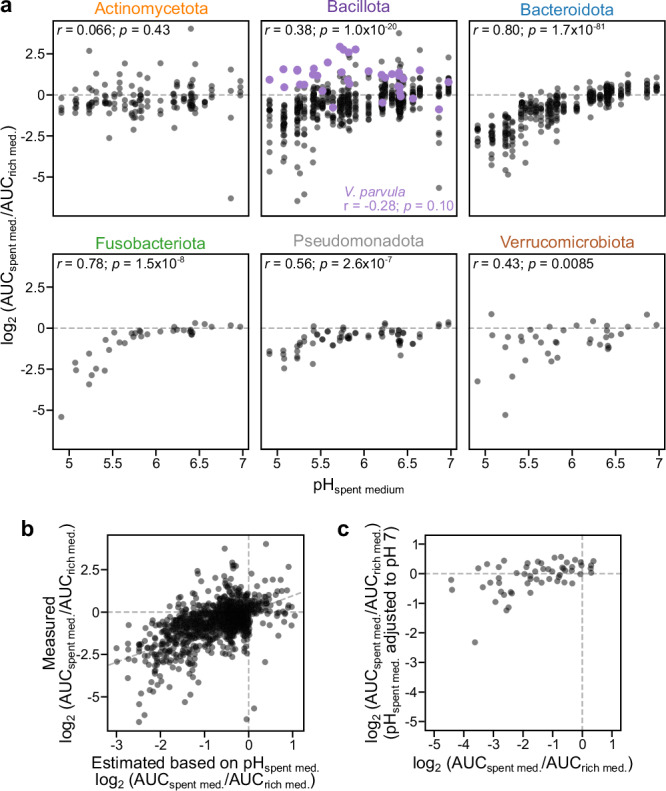
Negative growth interactions are correlated with lower environmental pH. **a** Pearson correlation of changes in growth in spent medium with the pH of the spent medium, separated by the phylum of the strain that was grown. **b** Estimation of growth effects of each strain in each spent medium based on a linear fit between growth effects and rich medium pH. **c** Comparison of growth effects in spent medium and the same spent medium adjusted to pH 7.

Since bacteria can modify and are affected by environmental pH, we studied how pH changes in the spent medium of one strain affected the growth of the cultivated strain. Overall, we observed a positive correlation between the pH of spent media (Supplementary Data 1) and the log_2_ fold change of growth in spent media compared to rich medium (*r*(1,222) = 0.49, *p* = 10^-73^, 95% CI [0.44, 0.53]; across all strain-spent media combinations), indicating that lower pH generally results in reduced growth. This correlation was particularly strong in the Bacteroidota, Fusobacteriota and Pseudomonadota phyla (*r* > 0.5; Supplementary Data 6), with the former two being more affected at lower pH (Fig. 3a). In contrast, the Actinomycetota phylum and some strains of the Bacillota phylum (e.g., *V. parvula*; Supplementary Fig. 3) showed weak or negative correlations. Similar results were observed when each strain was grown in rich medium adjusted to different pH values, relative to growth at pH 7 (Supplementary Fig. 3; Supplementary Data 7). Indeed, it was possible to reasonably predict the growth effect of each strain in each spent medium based on the medium pH alone (*r*(1,222) = 0.54, *p* = 10^-95^, CI [0.5, 0.58]; Fig. 3b and Supplementary Data 6).

To directly test whether changes in spent medium pH drove the observed growth differences, we selected 56 species pairs and measured their growth in spent medium adjusted to rich medium pH. As with replenished medium, most inhibitory interactions became neutral (Fig. 3c and Supplementary Data 8). Of note, replenishing the spent medium also increased its pH (Supplementary Data 1).

Overall, we observed that media acidification limits the growth of multiple strains. Although the degree of inhibition is strain-specific, some phyla both induce stronger pH reductions or exhibit greater sensitivity to acidic conditions.

### *V. parvula* possesses pH-neutralizing capabilities

**Fig. 4:**
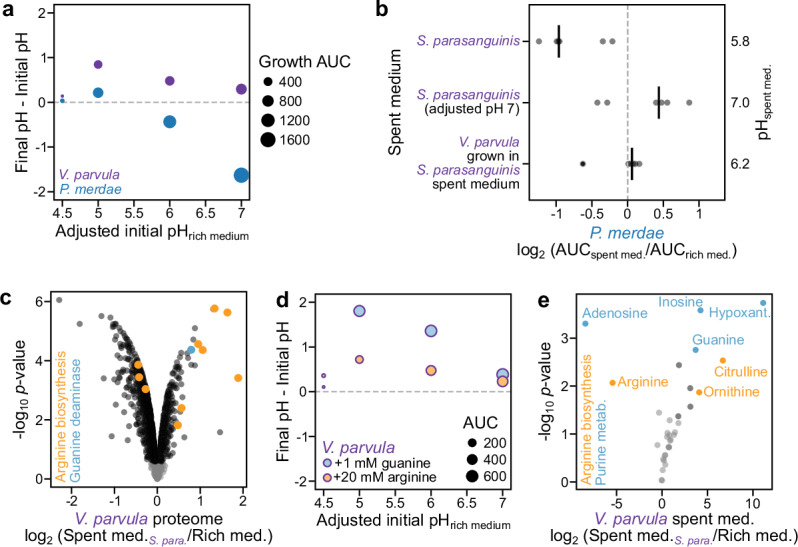
*Veillonella parvula* increases environmental pH and promotes the growth of low pH-sensitive strains. **a** Changes in pH of medium induced by the growth of *V. parvula* or *P. merdae,* depending on the initial pH of the rich medium. The size of points corresponds to the growth AUC in that condition and highlights that the lack of change in pH at the lowest pH is due to the absence of growth. **b** Growth of *P. merdae* when grown in *S. parasanguinis* spent medium, this spent medium adjusted to pH 7, or in the spent medium of *V. parvula* grown on *S. parasanguinis* spent medium. All conditions are compared to rich medium (data from individual six replicates and the median are shown), and the initial pH of spent medium is highlighted on the right. **c** Proteome abundance changes of *V. parvula* grown in *S. parasanguinis* spent medium compared to rich medium. Differential protein abundance was analyzed using limma empirical Bayes moderated two-sided *t* test, with Benjamini-Hochberg false discovery rate (FDR) correction for multiple comparisons. **d** As in panel (**a**) but with the addition of 1 mM guanine or 20 mM arginine prior to adjusting the pH of rich medium. **e** Metabolite level changes of *V. parvula* spent medium after being grown in *S. parasanguinis* spent medium compared to rich medium. Differential metabolite abundance was determined with a two-sided one-sample *t* test of log_2_ fold changes.

*V. parvula* showed a unique behavior compared to other bacteria, since its growth was promoted by 53% of the other strains (Fig. 1b). Further, it experienced the strongest growth stimulation when cultivated in the spent medium from *Bifidobacterium longum*, *Lacticaseibacillus paracasei*, *Streptococcus parasanguinis*, and *Clostridium perfringens*, all of which slightly acidified the pH of the medium to approximately 5.8 (Supplementary Data 1). This prompted us to examine if *V. parvula* possessed pH-neutralizing capabilities. To study this, we grew *V. parvula* in rich medium adjusted to varying initial pH, and measured the pH and overall growth after 48 h of incubation. As a comparison, we included *Parabacteroides merdae*, which displayed impaired growth at low pH and acidified the medium. The results showed that *V. parvula* increased the pH of the medium, with the strongest increase observed at pH 5, in which the final pH was raised by 0.85 units (Fig. 4a and Supplementary Data 9). Below pH 5, there was no bacterial growth. This pH-increasing effect gradually diminished as the initial pH increased from 5 to 7, suggesting that under acidic stress *V. parvula* attempts to neutralize the pH.

To test whether *V. parvula* could modify acidic spent media and make it more favorable for pH-sensitive strains, we incubated *P. merdae* (a pH-sensitive strain) in *S. parasanguinis* spent medium, which has a pH of 5.8. As expected, the growth of *P. merdae* was inhibited by 1.7-fold in *S. parasanguinis* spent medium when compared to rich medium (*t*(5) = − 4.7, *p* = 0.0052, 95% CI log_2_ fold change [− 1.2, − 0.36] on a two-sided one-sample *t* test), with this effect being alleviated when the pH of this spent medium was adjusted to pH 7 (*t*(5) = 1.3, *p* = 0.26, 95% CI log_2_ fold change [− 0.27, 0.79] on a two-sided one-sample *t* test; Fig. 4b and Supplementary Data 10). Similarly, *V. parvula* was able to modestly increase the pH of *S. parasanguinis* spent medium to 6.2, which rescued *P. merdae* growth to levels similar to rich medium (*t*(5) = − 0.31, *p* = 0.77, 95% CI log_2_ fold change [− 0.34, 0.27] on a two-sided one-sample *t* test). These results demonstrate that *V. parvula* can raise the pH of the media, thereby reducing acid-driven growth suppression of sensitive strains.

To investigate the mechanisms underlying this pH increase, we quantified the proteome of *V. parvula* grown in *S. parasanguinis* spent medium and in rich medium. This analysis showed an increased abundance of proteins involved in arginine biosynthesis, guanine deaminase (an enzyme that converts guanine to xanthine, releasing ammonia), and xanthine permease in the spent medium condition compared to the rich medium (Fig. 4c and Supplementary Data 11). Therefore, we tested if the addition of arginine or guanine to a rich medium adjusted to different pH would lead to increased final medium pH. It is important to note that pH was adjusted after the addition of guanine and arginine, potentially masking the pH-increasing effects of these molecules. While the addition of guanine led to a stronger increase in pH (by 1.8 units when starting at pH 5; Fig. 4d and Supplementary Data 9), the addition of arginine led to an effect similar to rich medium alone (compare Fig. 4d with Fig. 4a; increase by 0.72 units).

We then measured ammonia, amino acids, and nucleic acid components in the spent medium of *V. parvula* grown in *S. parasanguinis* spent medium compared to rich medium. Ammonia levels were not significantly increased (1.5-fold; *t*(2) = 3.0, *p* = 0.095, 95% CI log_2_ fold change [− 0.26, 1.5] on a two-sided one-sample *t* test), suggesting that it is not responsible for the pH increase. However, we observed changes in purine metabolism, with an increase of guanine, guanosine, adenine, hypoxanthine, and inosine, together with a decrease of adenosine (Fig. 4e and Supplementary Data 12). Further, arginine levels were lower, while citrulline, ornithine, aspartic acid, and glutamine were higher. This suggests that the proteome changes reflect the limited availability of some of these metabolites. Indeed, guanine supplementation increased growth of *V. parvula*, particularly at lower pH values (Fig. 4d and Supplementary Data 9). Given that *V. parvula* ferments organic acids for energy (e.g., lactate produced by *Streptococci*)^31^, the observed pH increase may reflect a reduction of extracellular organic acids.

In summary, *V. parvula* increased the pH of the extracellular environment, and this effect was exacerbated by guanine. This pH-neutralizing property can rescue the growth of species that are particularly affected by low pH (e.g., Bacteroidota).

### *V. parvula* pH-neutralizing effect plays an important role in community composition

**Fig. 5:**
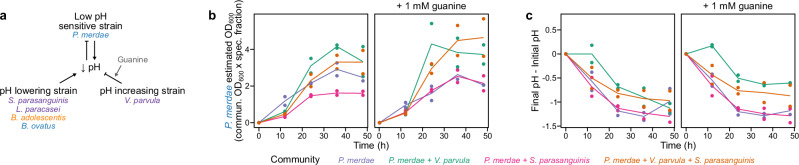
*Veillonella parvula* promotes the growth of a pH-sensitive strain in a community with a pH-lowering strain. **a** Model of contributing effects of each strain to the community. **b** *P. merdae* growth in different communities over time without or with the addition of guanine. Growth was calculated by multiplying the OD_600_ of the community by the fractional abundance of *P. merdae* in the community as determined by qPCR. **c** Environmental pH of different cultures over time.

We pondered if the capacity of *V. parvula* to neutralize the pH of the medium would play a role in a community setting. Thus, we assembled three-member communities consisting of *V. parvula, P. merdae* (a pH-sensitive strain), and an acidifier strain (Fig. 5a). We chose four phylogenetically diverse acidifying strains: *S. parasanguinis* and *Lacticaseibacillus paracasei* from the Bacillota phylum, *Bifidobacterium adolescentis* from the Actinomycetota phylum, and *Bacteroides ovatus* from the Bacteroidota phylum. In all co-cultures with acidifying strains, *P. merdae* exhibited reduced growth and a lower pH compared to its monoculture (Fig. 5b, c, Supplementary Fig. 4 and Supplementary Data 13). *B. ovatus* growth rate was slightly higher than *P. merdae* (Supplementary Fig. 4e and g), resulting in a stronger decrease in pH than the other acidifying strains (Supplementary Fig. 4f), both of which prevented *P. merdae* from growing in co-culture. The addition of *V. parvula* to these communities consistently enhanced *P. merdae* growth and elevated the pH (Fig. 5b and Supplementary Fig. 4), demonstrating the capacity of *V. parvula* to increase the environmental pH in diverse community settings and promote the growth of low pH-sensitive strains.

Since we identified that guanine strengthens the pH-increasing capacity of *V. parvula*, we evaluated its role in these communities (Fig. 5a). As expected, the addition of guanine promoted *P. merdae* growth and increased the pH in communities in which *V. parvula* was present (Fig. 5b and Supplementary Fig. 4) except for *B. ovatus* communities due to the aforementioned reasons.

In conclusion, *V. parvula* supports the growth of strains sensitive to low pH in a community, with this effect being amplified by the addition of guanine.

## Discussion

We systematically measured all mutual growth interactions between a group of representative strains of the human gut microbiome. For scalability, the analysis was limited to pairwise interactions, since testing all possible community compositions of our group of 36 strains would require testing over 10^10^ conditions^19^. Pairwise interactions generally enable predicting the overall composition of a community^12,14^. However, emergent behaviors of higher-order interactions are possible^32^, as evidenced by our communities with *P. merdae* and *B. ovatus*, and warrant further study. We used a spent medium approach, which disregards contact-dependent mechanisms^33^, but facilitates growth measurements. It also eases mechanistic follow-up work, particularly when using an approach based on proteomics, due to the high dynamic range of the proteome and the general dominance of one strain in co-cultures^16^, or metabolomics, due to the difficulty in identifying which strain consumed or produced a metabolite^14^. In this work, all experiments were performed in rich medium, which neglects specific nutrients that are present in the gut (e.g., complex carbohydrates), and interactions with the host (both the environment it provides, and how it reacts to the presence of microbes). Yet, our approach is applicable to other media and could enable the discovery of further interaction mechanisms in the future.

To quantify interspecies effects, we employed a simple metric (area under the growth curve) that integrates growth rate and overall biomass yield. However, we provide time-resolved growth measurements for all experiments. These can be visually inspected or fitted to growth models to identify the cause of changes to the area under the curve. Further, growth parameters can be integrated with computational approaches to predict community dynamics more accurately. Incorporating metabolic modeling^21,34^ may further illuminate the mechanistic basis of the observed interactions.

Our results highlighted a general prevalence of negative interactions, in accordance with previous studies^2,35^. Strains tended to grow more poorly on spent media derived from closely related taxa, which could reflect competition for similar metabolic niches. Yet, we observed that for multiple strain pairs, the low pH of the spent medium was sufficient to limit growth^10–12,36–38^. While bacteria form niches in the gut^39^, it remains to be seen if this spatial segregation is sufficient to create local pH environments or if they are buffered by the gut. Thus, it is important to distinguish inhibitory interactions that are not driven by pH changes. For example, we observed that *B. fragilis* and *B. uniformis* spent media inhibited the growth of enterotoxin-producing *B. fragilis* (*B. fragilis* ET) and *A. muciniphila* strains, respectively, even after adjusting its pH to neutral. In both cases, the inhibition also persisted in replenished medium, suggesting that the former strains produce factors that inhibit the growth of the latter ones. Of note, *A. muciniphila* growth was inhibited by the spent and replenished media of other Bacteroidota species, which could warrant further study, given that these species share a spatial niche (the mucus layer).

Other specific interactions identified in our screening approach are in line with effects that have previously been observed in vivo. For example, we detected a strong inhibitory effect of *Clostridioides difficile* spent medium across multiple strains (32% of the strains showed a 1.5-fold lower growth in *C. difficile* spent medium compared to rich medium), in agreement with the role of this opportunistic pathogen in decreasing microbiome diversity^40^. We also identified that *M. gnavus* growth can be promoted by vesicles from another species of the Clostridia class. *M. gnavus* is known to exchange DNA with other members of the same species via membrane vesicles^41^, it is possible that this extends to other related species.

Our study highlighted the capacity of *Veillonella parvula* to increase environmental pH and facilitate the growth of low pH-sensitive strains. This could explain the role in communities for this prevalent ( > 50% of human gut microbiomes contain this species^22,23^), but low-abundant species (generally < 1% abundance in gut microbiomes^22,23^, and 2–8% in our simple communities). The pH-increasing capacity of this species was enhanced by the addition of guanine, which also promoted its growth. Thus, the upregulation of guanine deaminase and of the arginine pathway when *V. parvula* was grown in *S. parasanguinis* spent medium could pinpoint a limited access to these metabolites. In line with this, *S. parasanguinis* is known to be strongly arginolytic^25,42^.

In summary, we provide a comprehensive dataset of interactions among human-relevant gut bacteria. Our approach to suggest interaction mechanisms at the molecular level, by measuring proteome or metabolome changes of one strain in the spent medium of another, compared to rich medium, can be applied to other identified interactions. Understanding the mechanisms behind interactions can enable strategies for the rational manipulation of gut microbial communities.

## Methods

### Spent medium assay

Spent media was prepared by growing each strain in Gifu Anaerobic Medium (GAM) under anaerobic conditions at 37 °C for 24 h inside a Coy anaerobic chamber (5% H_2_, 10% CO_2_, 85% N_2_). Cultures were centrifuged at 2000 × *g* for 30 min at 4 °C, and the supernatant was filtered through a 0.2 µm membrane filter (Filtropur S, Sarstedt) to remove cells. The resulting cell-free supernatant (spent medium) was used immediately for growth assays. For growth assays, 150 µL of medium, either standard GAM, spent medium, or a replenished medium composed of 50% spent medium and 50% 2 x concentrated GAM, was added to each well of a 96-well plate. To this, 50 µL of a diluted overnight bacterial culture was added, resulting in a final OD_600_ of 0.01. Each condition was tested at least in duplicate. Plates were sealed with a breathable membrane (Breathe-Easy, Diversified Biotech) and incubated at 37 °C in a Coy anaerobic chamber, and OD_600_ was recorded every 3 min for 48 h using a Cerillo Stratus plate reader under static conditions. We confirmed that for all strains the growth measurements were similar if performed under shaking conditions at 375 r.p.m. (*r*(32) = 0.97, *p* = 10^−20^, 95% CI [0.94, 0.98]; Supplementary Fig. 5a and Supplementary Data 14).

### Growth assay data analysis

Growth curves were first trimmed at strain-specific time points when they reached early stationary phase to avoid diluting growth responses. The same time point was used for all experiments of the same strain (Supplementary Data 1). All growth curves were then visually inspected, and replicates where the rich medium condition deviated from the overall behavior of that strain in rich medium (observed in all other experiments) were removed. In those cases, all conditions of that replicate (rich medium, spent medium, and replenished media) were removed. The area under the growth curve (AUC) was then calculated using the trapezoid method, and fold changes to the rich medium condition were calculated. In rare cases in which the AUC was negative in spent or replenished media, due to lack of growth or growth below the detection limit of the plate reader, the AUC was set to the lowest AUC value for that strain in the respective media (so that a fold-change could be calculated). Performing the same analysis using the average OD_600_ within a 30 min window around the trimming time point resulted in similar results to using the AUC (Supplementary Fig. 5b; *r*(2,416) = 0.92, *p* = 10^−959^, 95% CI [0.91, 0.92]). We opted for AUC to reflect both changes in yield and growth rate. For all follow-up experiments, the spent medium assay was repeated in that specific condition.

### Proteomics analysis

Bacterial cells were harvested in triplicate by centrifugation at 3000 × *g* for 15 min at 4 °C from a 24 h culture. This timepoint was chosen since we observed a change in final OD_600_, and expected that molecular changes would reflect the status of the cell that led to the growth changes. The pellet was resuspended in PBS containing 2% SDS and incubated at 98 °C for 10 min. Protein concentration was determined using the BCA assay. 6 µg of total protein from each sample was denatured with a final concentration of 2% SDS and 20 mM TCEP. Samples were digested with a modified sp3 protocol^43,44^. Samples were added to a bead suspension (10 μg of beads; Sera-Mag Speed Beads, 4515-2105-050250, 6515-2105-050250, in 10 μl 15% formic acid and 30 μl ethanol) and incubated shaking for 15 min at room temperature. Beads were then washed four times with 70% ethanol. Proteins were digested overnight by adding 40 μl of 5 mM chloroacetamide, 1.25 mM TCEP, and 200 ng trypsin in 100 mM HEPES pH 8.5. Peptides were eluted from the beads and dried under vacuum prior to labeling with tandem mass tags (Thermo Fisher Scientific). Labeled peptides were pooled and desalted with solid-phase extraction using a Waters OASIS HLB μElution Plate (30 μm). Samples were fractionated into 48 fractions on a reversed-phase C18 system running under high pH conditions, with every sixth fraction being pooled together. Samples were analyzed on a Thermo Fisher Scientific Vanquish Neo LC coupled with a Thermo Fisher Scientific Orbitrap Exploris 480 using a Nanospray-Flex ion source and an emitter with dimensions 360 μm OD × 20 μm ID; 10 μm tip. The mass spectrometer was operated in positive ion mode with a spray voltage of 1.9 kV and capillary temperature of 274 °C. Full-scan mass spectrometry spectra with a mass range of 375–1500 m/z were acquired in profile mode using a resolution of 60,000 (normalized automatic gain control target at 300%). Fragmentation was triggered for the top peaks within a 2 s window with charge 2-6 on the MS scan (data-dependent acquisition) with a 30 s dynamic exclusion window (normalized collision energy was 38), and MS/MS spectra were acquired in profile mode with a resolution of 45,000 (normalized automatic gain control target at 200%). Raw files were processed with MSFragger v3.8^45^ against the UniProt proteome of the grown and spent medium strains (UP000000818 and UP000004410 for the *M. gnavus* grown in *C. perfringens* spent medium experiment, and UP000778864 and UP000001502 for the *V. parvula* in *S. parasanguinis* spent medium experiment). Spectra were searched with tryptic specificity (cleavage after K/R, excluding KP/RP sites), allowing up to two missed cleavages. Precursor and fragment mass tolerances were set to 20 ppm. Carbamidomethylation of cysteine ( + 57.0215 Da) and TMT labeling of lysine residues were specified as fixed modifications, while methionine oxidation ( + 15.9949 Da), TMT labeling of peptide N-termini ( + 229.1629 Da) and protein N-terminal acetylation ( + 42.0106 Da) were included as variable modifications (maximum three variable modifications per peptide). Peptides were required to be 7-50 amino acids in length. Peptide-spectrum matches, peptides, and proteins were filtered to a 1% false discovery rate (FDR) using a target-decoy strategy, and proteins were reported when supported by at least two unique peptides. The median abundance in each TMT channel was normalized to the median intensity of all TMT channels, and statistical significance was determined using limma^46^.

### Growth assays for measuring the effect of nucleotides on *M. gnavus*

Adenine, guanine, uracil, and xanthine were dissolved in 0.1 M NaOH and added to rich medium at a final concentration of 1 mM. The addition of NaOH did not significantly alter the pH; a rich medium containing the same volume of NaOH was used as a control. D-ribose was dissolved in water and added to rich medium at a final concentration of 1 mM. Adenosine, cytidine, guanosine, 2-deoxythymidine were dissolved in PBS and added to the rich medium at a final concentration of 1 mM. DNA (low molecular weight from salmon sperm) and RNA (from yeast) were dissolved in PBS and added to rich medium at a final concentration of 0.5 mg/mL. *M. gnavus* was inoculated into each condition at an initial OD_600_ of 0.01, and cultures were incubated anaerobically at 37 °C. Growth was monitored for 48 h as described above, with data analyzed in a similar manner.

### Crude extracellular vesicle isolation and growth assays

Vesicles were isolated from 500 mL of *Clostridium perfringens* culture grown anaerobically in rich medium for 24 h. Cultures were first centrifuged at 2000 × g for 30 min at 4°C to remove bacterial cells. The resulting supernatant was sterilized by filtration through a 0.2 µm membrane filter (Thermo Fisher Scientific 0.2 aPES) and subsequently ultracentrifuged at 100,000 × *g* for 4 h at 4 °C using a Ti 45 rotor (Beckman Coulter Optima) to pellet crude extracellular vesicles. The pellet was resuspended in 1 mL PBS and stored at − 20 °C until further use. To assess the effects of vesicles and related fractions on *M. gnavus* growth, isolated vesicles were diluted 1:50 in GAM prior to testing. *C. perfringens* spent medium (from which the vesicles were isolated), and vesicle-free supernatant (the supernatant after ultracentrifugation) were tested without dilution. Boiled versions of crude vesicles, spent medium, and the vesicle-free supernatant were prepared by incubating each sample at 98 °C for 10 min. *M. gnavus* was inoculated into each condition at an initial OD_600_ of 0.01, and cultures were incubated under anaerobic conditions at 37 °C. Growth was monitored for 48 h as described above, with data analyzed in a similar manner.

### Dynamic light scattering analysis of extracellular vesicles

The size of extracellular vesicles was determined using dynamic light scattering (DLS) with a Malvern Zetasizer Nano ZS at 25 °C. The vesicle samples were diluted two-fold in PBS, and all other samples were analyzed without dilution. Three biological replicates of each sample were independently prepared, and each one was measured three consecutive times and averaged.

### Negative staining transmission electron microscopy of extracellular vesicles

Vesicles and particles from rich medium were imaged using negative staining transmission electron microscopy. For this, formvar-carbon-coated grids were glow-discharged for 30 s. The sample was incubated on the grid for 1 min. Excess sample was blotted off with blotting paper. Grids were then washed with a drop of water, followed by negative staining with 1.5% uranyl acetate for 1 min. The grids were then dried out. Imaging was performed using a Thermo Fisher Scientific Talos L120C operating at 120 kV. Digital images were acquired with the Velox software v2.16 using an integrated CCD camera system.

### Growth of *V. parvula* and *P. merdae* at different pH and supplemented with guanine and arginine

Stocks of guanine (1 mM, prepared by dissolving in 1 M NaOH) and arginine (20 mM, prepared by dissolving in sterile water) were prepared. These stocks were first added to one-third of the required volume of 2 x GAM by carefully dropping under slow stirring. The pH of GAM, GAM supplemented with guanine, or GAM supplemented with arginine was then adjusted to 4.5, 5, 6, or 7 using 1 M HCl and filter sterilized. The mixture was transferred to an anaerobic chamber, where two-thirds of 2 x GAM was added, and finally diluted to the desired volume with sterile double-distilled water. The final media were incubated in an anaerobic chamber at least 12 h before use. *V. parvula* was grown overnight in GAM supplemented in 0.5% lactate to improve growth. *V. parvula* and *P. merdae* were inoculated into each condition at an initial OD_600_ of 0.01, and cultures were incubated under anaerobic conditions at 37 °C. Growth was monitored over a 48 h period as described above, with data analyzed in a similar manner.

### Quantification of amino acids and nucleic acid components in spent media

Amino acids and nucleic acid components were quantified by liquid chromatography coupled to mass spectrometry. For amino acids, these were extracted from spent media by mixing 100 µL spent media from three cultures with 400 µL acetonitrile. Samples were centrifuged at 4 °C, 10,000 × *g* for 10 min. The supernatant was diluted 10 and 100 times for amino acid and ammonia analysis, respectively. An extraction blank was prepared in the same way as the samples, using 100 µL water. Extracted samples were derivatized by AccQ-Tag™ (Waters) by mixing 20 µL of diluted supernatant with 60 µl AccQ-Tag Ultra Borate buffer, including internal standards for amino acids followed by the addition of 20 µl AccQ-Tag derivatization solution. Samples were vortexed, kept at room temperature for 10 min followed by 10 min at 55 °C. Calibration curves were prepared in the same way as the samples. Derivatized samples contained 0.5 pmol/µL of internal standards. For nucleic acid components, spent media (100 µL) was extracted with acetonitrile (400 µL). Extracts were centrifuged for 10 min at 6100 × *g* at 4 °C. Supernatant (50 µL) was collected and dried in a 96-well plate under nitrogen flow at 40 °C. Upon analysis, samples were resuspended with 20 µL 0.1% FA in 5% methanol.

Samples were analyzed using an Agilent 1290 Infinity UHPLC system coupled to an Agilent 6495D triple quadrupole mass spectrometer equipped with a jet stream electrospray source operating in positive or negative ion mode. For amino acids, separation was achieved on a Waters BEH C18 2.1 × 100 mm, 1.7 μm column held at 50 °C, while for nucleic acid components, a Waters Acquity UPLC HSS-T3 50 × 2.1 mm, 1.8 µm column held at 40 °C was used. Mobile phases consisted of 0.1% formic acid in water (A) and 0.1% formic acid in acetonitrile (B). For amino acids, the following gradient was used 0-0.54 min (0% B at 0.5 mL/min), 3.5 min (9.1% B at 0.5 mL/min), 7 min (17% B at 0.5 mL/min), 8 min (19.7% B at 0.5 mL/min), 8.5 min (19.7% B at 0.5 mL/min), 9 min (21.2% B at 0.5 mL/min), 10 min (59.6% B at 0.5 mL/min), 11 min (95% B at 0.5 mL/min), 11.5 min (95% B at 0.5 mL/min) and conditioning up to 5 min (0.1% B at 0.8 ml/min). For nucleic acid components, the gradient was 0-0.4 min (0.1% B at 0.3 mL/min), 1.6 min (7% B at 0.3 mL/min), 2.5 min (30% B at 0.4 mL/min), 2.8 min (90% B at 0.6 mL/min), 3.2 min (80% B at 0.5 mL/min), and conditioning up to 5 min (0.1% B at 0.5 mL/min).

Mass spectrometry parameters were optimized for each compound (Supplementary Data 15). The fragmentor voltage was set at 166 V, the cell accelerator voltage at 7 V (for amino acids) or 2 V (for nucleic acid components) and nitrogen was used as collision gas. Jet-stream gas temperature was 290 °C at 11 L/min (for amino acids) or 200 °C at 12 L/min (for nucleic acid components). Sheath gas temperature was 325 °C (for amino acids) or 280 °C (for nucleic acid components) at 12 L/min. The nebulizer pressure was set to 20 psi and the capillary voltage was set at 4 kV (for amino acids) and 3 kV (for nucleic acid components).

Peaks were integrated and processed using Agilent MassHunter Quantitative Analysis for QqQ, v12.0. Peak area was normalized by OD_600_ at the time of collection of spent media. Statistical significance was determined with a two-sided one-sample *t* test of log_2_ fold changes.

### Community assembly and growth conditions

Microbial communities consisting of two or three bacterial strains (*P. merdae, V. parvula, S. parasanguinis, L. paracasei, Bifidobacterium adolescentis, and Bacteroides ovatus*) were assembled and cultured under two conditions: GAM and GAM supplemented with 1 mM guanine. Following guanine addition, the pH of the supplemented medium was adjusted to match that of the standard GAM to ensure consistency across conditions. Each strain was inoculated into the respective medium at an initial OD_600_ of 0.01. Cultures were incubated under anaerobic conditions at 37 °C, and OD_600_ was measured at 12, 24, 36, and 48 h. At each time point, a volume of sample corresponding to one unit of OD_600_ was collected. Cells were harvested by centrifugation, and the resulting pellets were stored at − 20 °C for downstream analyses.

### Quantitative polymerase chain reaction (qPCR)

Genomic DNA was extracted from bacterial community cultures using the MP FastDNA™ kit (MP Biomedicals) according to the manufacturer’s instructions. DNA concentration and purity were assessed using a NanoDrop spectrophotometer (Thermo Fisher Scientific). PCR was performed on a Bio-Rad CFX Duet real-time PCR system using 2x iQ™ SYBR® Green Supermix (Bio-Rad). Each 10 µL reaction contained 1x iQ SYBR Green Supermix, 0.2 µM of each primer (sequences in Supplementary Data 16; purchased from Integrated DNA technologies), and 1 ng of template DNA, with nuclease-free water added to the final volume. The thermal cycling conditions were: initial denaturation at 95 °C for 3 min, followed by 35 cycles of denaturation at 95 °C for 15 s, annealing at 59 °C for 30 s and extension at 72 °C for 30 s. Absolute gene copy numbers were determined using a standard curve generated from serial 10-fold dilutions of custom-made plasmids containing the target genes (10^2^–10^8^ copies per reaction). Absolute copy numbers were corrected by the gene copy number in the genome. The fraction of each species in the community was calculated as the ratio of the corrected copy number of that species to the summed corrected copy numbers of all species present in the community. OD_600_ for each species was estimated as the total OD_600_ of the community multiplied by the fraction of the species.

### Statistics and reproducibility

No statistical method was used to predetermine sample size. No data were excluded from the analyses, apart from what is described under Growth assay data analysis. The experiments were not randomized. The Investigators were not blinded to allocation during experiments and outcome assessment.

### Reporting summary

Further information on research design is available in the Nature Portfolio Reporting Summary linked to this article.

## Supplementary information

Supplementary Information

Description of Additional Supplementary Files

Supplementary Data 1

Supplementary Data 2

Supplementary Data 3

Supplementary Data 4

Supplementary Data 5

Supplementary Data 6

Supplementary Data 7

Supplementary Data 8

Supplementary Data 9

Supplementary Data 10

Supplementary Data 11

Supplementary Data 12

Supplementary Data 13

Supplementary Data 14

Supplementary Data 15

Supplementary Data 16

Reporting Summary

Transparent Peer Review file

## Data Availability

Raw growth data can be found at https://zenodo.org/records/17456178 or in the supplementary data. The mass spectrometry proteomics data have been deposited to the ProteomeXchange Consortium via the PRIDE partner repository with the dataset identifier PXD070029. The mass spectrometry metabolomics data have been deposited to MassIVE with the dataset identifier MSV000102556.

## Code availability

The code to reproduce the growth assay data analysis is available at https://github.com/mateuslab-prot/SpeciesSpeciesInteractions (10.5281/zenodo.21494737).
