# Supplementary material for: Systematic profiling of growth interactions in human gut microbiome species
Source: Nat Commun. 2026 Aug 7;17:8012. doi: 10.1038/s41467-026-76526-z (PMC13451373; doi:10.1038/s41467-026-76526-z)
Supplement: Supplementary file 2 [file 41467_2026_76526_MOESM1_ESM.pdf]

Supplementary information

Systematic profiling of growth interactions in human gut microbiome species

Bolor Buyanbadrakh, Elisabeth Baland, Paula Lambeck, Lucía Pérez Jiménez, Sandra M. Holmberg, Fabiola Puértolas-Balint, Eric Toh, Sun Nyunt Wai, Björn O. Schröder, Madeleine Ramstedt, Shaochun Zhu, André Mateus

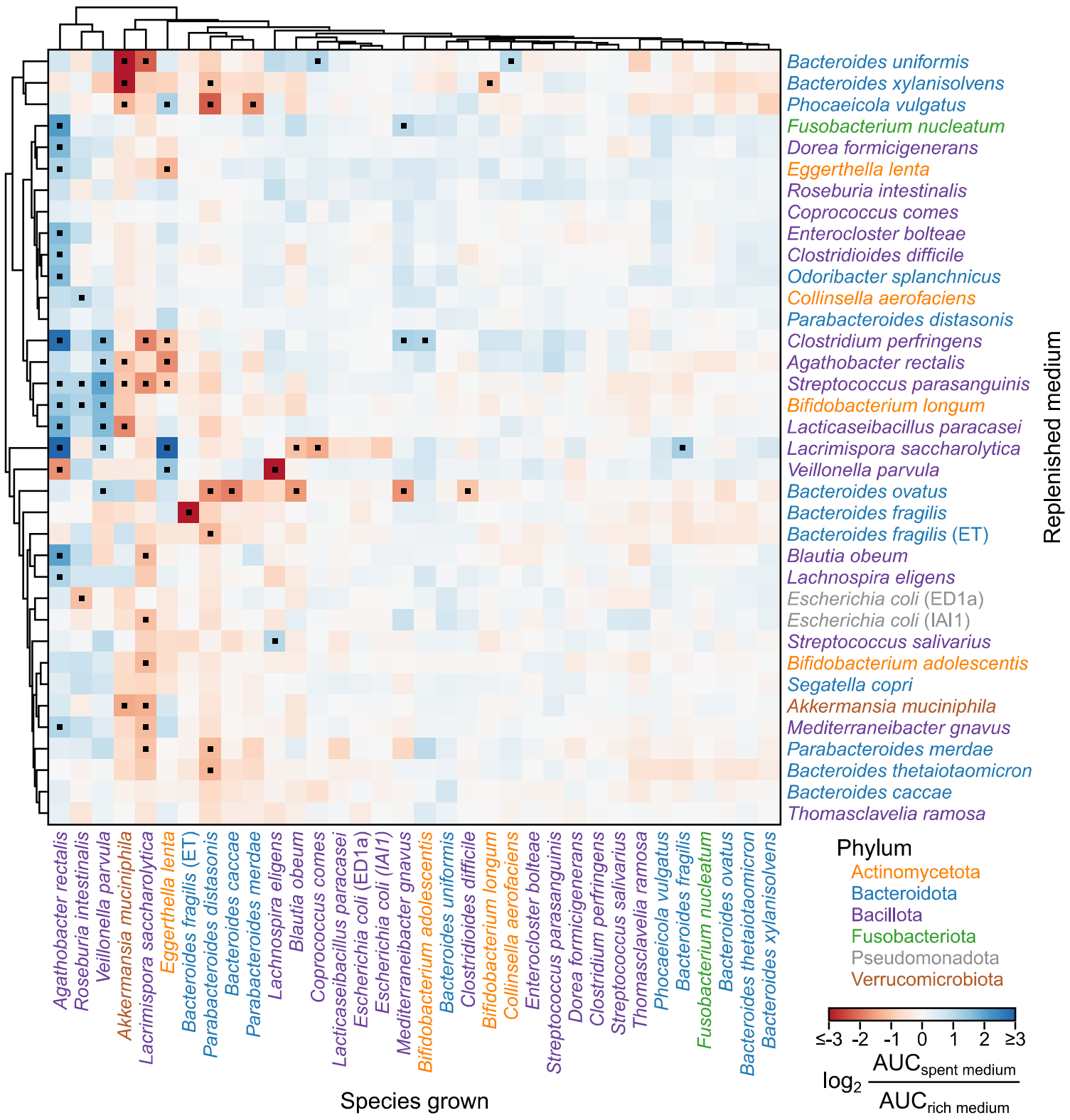

Supplementary Fig. 1. Pairwise changes in AUC of strains grown (column) in replenished medium (row) of another strain compared to rich medium. Strains are color coded according to the phylum to which they belong. Cases in which the absolute fold change is >2 are marked in black.

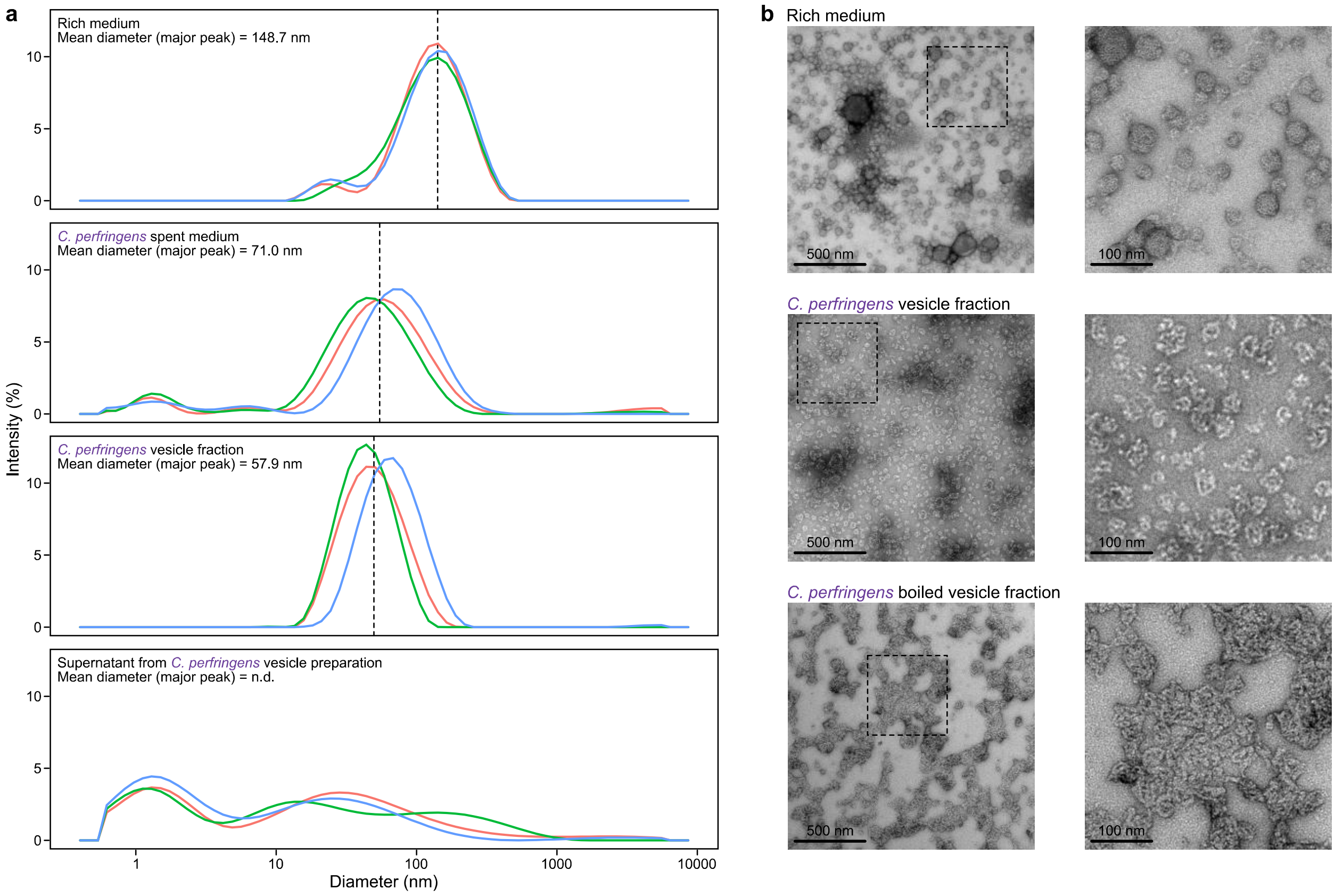

Supplementary Fig. 2. Characterization of particles in rich medium and *C. perfringens* spent medium. (a) Particle size distribution using dynamic light scattering. **(b)** Negative-stain electron microscopy. A single replicate of the experiment was performed.

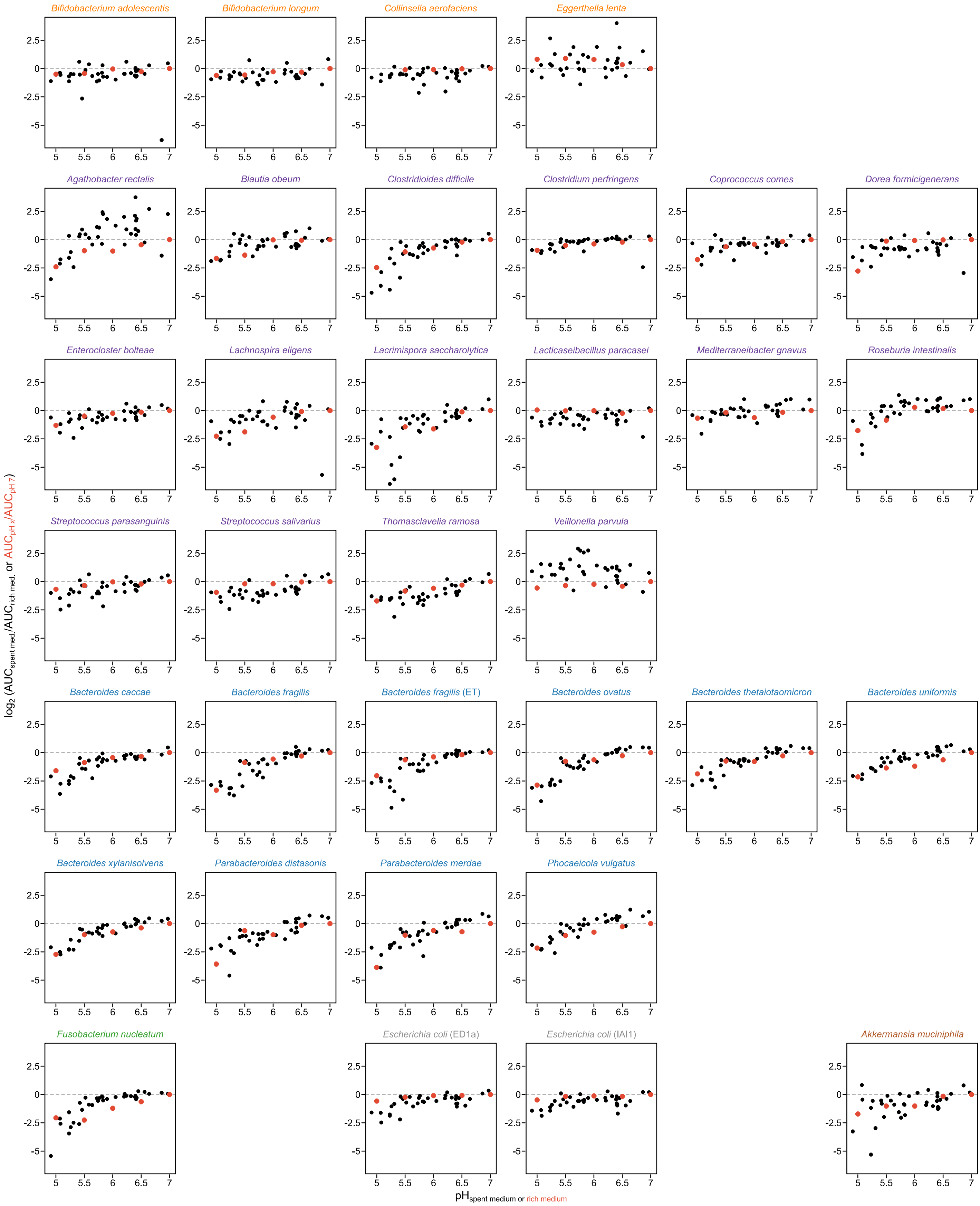

Supplementary Fig. 3. Correlation between medium pH and growth. Strain-wise comparison of changes in growth in spent medium (black) or rich medium at a defined pH (red).

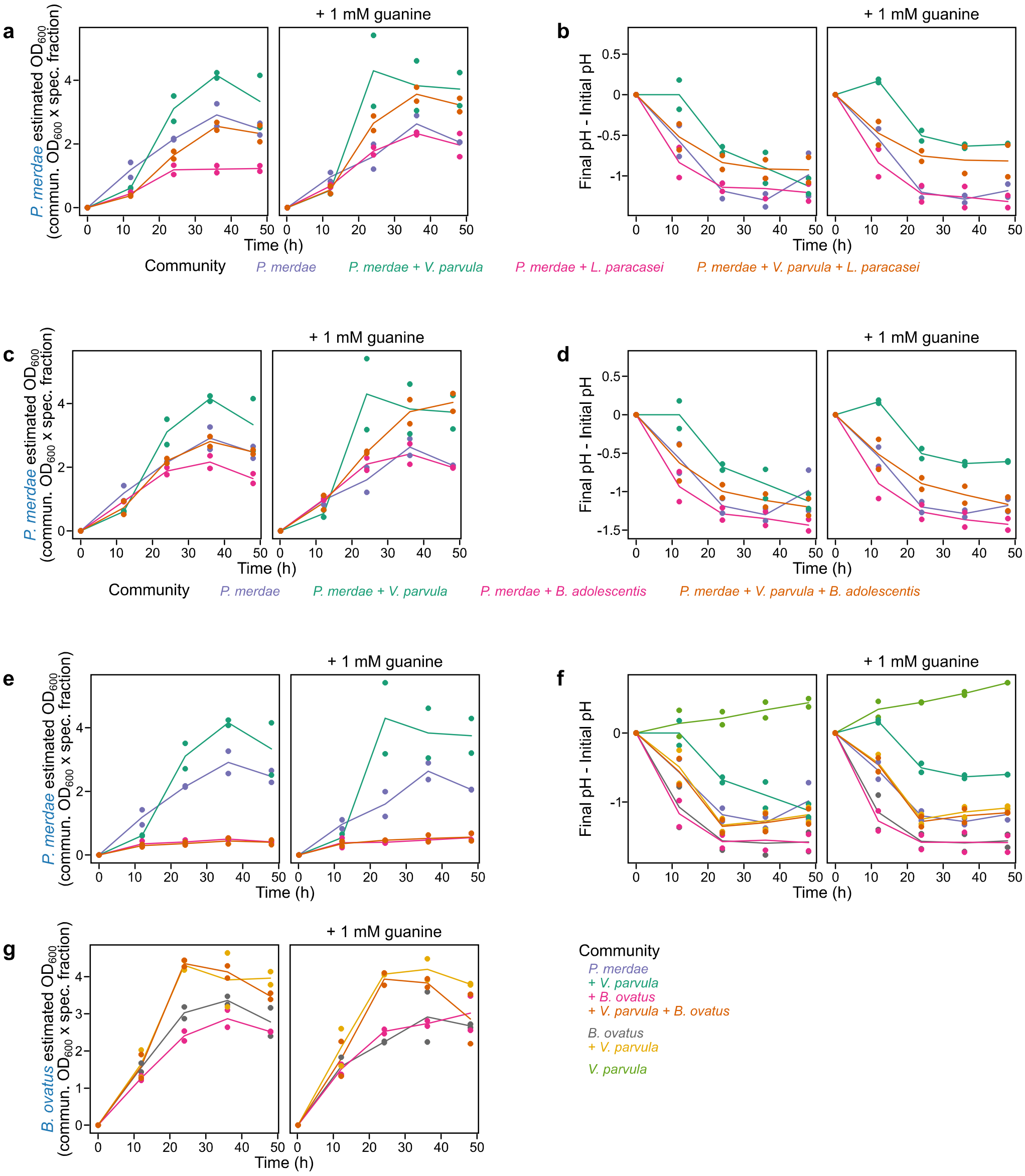

Supplementary Fig. 4. Effect of *V. parvula* on the growth of *P. merdae* and pH in bacterial communities with (a-b) *L. paracasei*, **(c-d)** *B. adolescentis*, and **(e-f)** *B. ovatus* (as in Error! Reference source not found.**b**). **(g)** *B. ovatus* growth in the latter community.

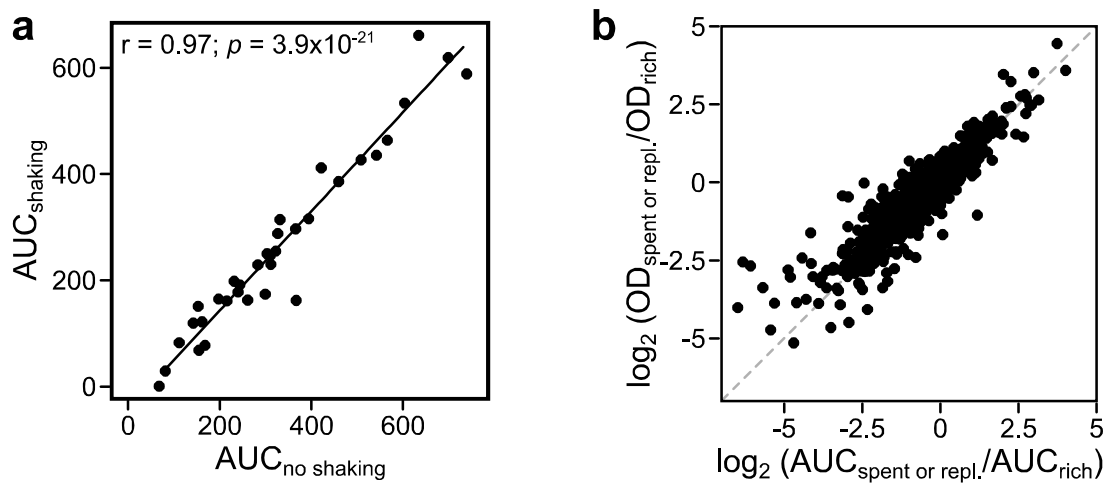

Supplementary Fig. 5. Effect of shaking and metric for growth measurements. (a) Correlation between AUC determined when growth measurements were performed under shaking conditions or not. **(b)** Correlation between using AUC or OD_{600} as the growth metric.
