# Supplementary material for: Systematic profiling of growth interactions in human gut microbiome species
Source: Nat Commun. 2026 Aug 7;17:8012. doi: 10.1038/s41467-026-76526-z (PMC13451373; doi:10.1038/s41467-026-76526-z)
Supplement: Supplementary file 3 [file 41467_2026_76526_MOESM20_ESM.pdf]

nature portfolio

Peer Review File

Systematic profiling of growth interactions in human gut microbiome species

Corresponding Author: Dr Andre Mateus

This file contains all reviewer reports in order by version, followed by all author rebuttals in order by version.

Version 0:

Reviewer comments:

Reviewer #1

(Remarks to the Author)
The manuscript by Buyanbadrakh et al. investigates pairwise interactions among 36 representative gut bacterial strains, reporting that approximately 25% of interactions result in growth inhibition and 4% lead to growth promotion. The authors also provide additional insights into specific interactions, particularly those between M. gnavus and C. perfringens, and between Veillonella and P. merdae. Overall, the manuscript is clearly written, the data is well presented, and the figures are of high quality. The methods are mostly sound, and the selection of representative strains is reasonably justified. The incorporation of proteomic analyses of selected bacteria grown in spent versus fresh media is a strength.
However, I remain uncertain about the ecological significance of the findings, and the extent to which they advance our understanding of these interactions is somewhat limited, as I outline in my comments below. Despite these limitations, the study provides a systematic dataset of interactions between representative gut bacteria. This offers a useful foundation for future efforts to analyse microbe-microbe interactions.

1. Many of the reported interactions appear to be driven largely by acidification of spent media. It is unclear how relevant these pH shifts are for bacterial interactions in vivo, given that distal gut pH is generally stable and any local drops are expected to be transient? Therefore, I would have expected that the interactions/growth in spent media were tested after adjusting the pH of spent media. Indirectly adjusting pH by diluting with rich media, as the authors did, does not allow to disentangle the effect of cross-feeding/nutrient depletion/inhibitory metabolites vs. pH, as it also dilutes spent media. Unless the authors can convincingly discuss the ecological relevance of such changes in pH, further experiments on how pH really affects results would be important, i.e. testing interactions with spent media with pH adjusted only (no/minimal dilution).

1a. Lines 68-70: would be good to see the heatmap equivalent to Fig.1b when the spent medium was replenished with nutrients. For instance, do interactions between M. gnavus and perfringens still hold?

2. The OD measurements were performed using a Cerillo Stratus plate reader, which does not incorporate shaking. This raises concerns about the accuracy of OD readings, especially for strains that tend to aggregate or form biofilms. Without agitation, cells can rapidly settle, leading to inconsistent or misleading OD values.
The uthors present in Fig.1c the replicate variability (I commend the authors for reporting this), but does one know whether the device reliably captured cells in suspension rather than reading mostly settled cultures?

3. The authors nicely show that vesicles produced by C. perfringens boost M. gnavus growth, and literature supports the presence of nucleic acids in these vesicles - but addition of nucleic acids does not boost M. gnavus growth - why? How do authors envision that vesicles containing DNA would?

4. For the proteomics analysis, bacterial cells were harvested after 24 h of growth (methods). Is 24 hours an appropriate time point to capture biologically relevant differences in the proteome? Table S1 suggests cultures would be already in the stationary phase at 24h, for both M. gnavus and V. parvula.

5. The mechanism via which Veilonella is increasing pH should be further explored: ammonia production would be an obvious one, as levels of the enzyme guanidine deaminase are increased (Fig.4c), and this is known to catalyse the hydrolytic deamination of guanine to xanthine and ammonia. Has ammonia production been measured?

6. The discussion lacks depth in addressing how the results from these in vitro pairwise assays can be generalized to community level dynamics under the physiological conditions of the gut. A broader consideration of how these findings translate to the complex, spatially structured, mostly buffered environment of the intestine is missing.

In addition, some statements are insufficiently explained. For instance: "M. gnavus produces an anti-C. perfringens bacteriocin emphasizing strong growth dynamics between these species." What do the authors implicate by this, I do not see any inhibition of perfringens growth in M. gnavus spent media. Is this because a different strain was used?

Other points:

Results, lines 74-78: authors start the paragraph with "We next explored if inhibitory interactions were mostly reciprocal…". The way it is currently written, it gives the impression that all percentages reported are a % of inhibitory interactions, not of the total interactions tested. This needs to be rephrased for clarity.

(Remarks on code availability)

Reviewer #2

(Remarks to the Author)
Buyanbadrakh et al profile the pairwise interactions between 36 gut bacterial strains using a spent medium assay. The authors report that most interactions are inhibitory and due to resource depletion or medium acidification. The authors then focus on a few positive interactions to investigate mechanisms. The dataset is valuable and the follow-up mechanistic experiments on a few selected interactions are interesting. My main comments are about clarifying how interactions are quantified, tone down a few general claims so they more closely match the experiments and data, and add quantitative values/ statistics when missing. See my comments below for details.

Main comments:

1. The authors state that "Most interactions were inhibitory and largely explained by resource depletion or medium acidification." (L19) and "Widespread growth inhibiting and growth promoting ecological interactions among human gut microbes." (Figure 1 legend; L94). However, Figure 1d does suggest that most interactions are actually neutral under the 2-fold change threshold. I think that the statement that "Most interactions were inhibitory" should be clarified and rephrased.
It would also be helpful to show the following heatmaps: a heatmap like Fig 1b for the replenished medium data and heatmaps with the 2-fold change threshold for both the spent medium and the replenished medium. This would make it clearer which pairwise interactions are inhibitory and more likely driven by resource depletion vs other mechanisms of inhibition, and which interactions are neutral or positive.

2. L60. "Growth dynamics were quantified by calculating the area under the growth curve (AUC)." Because this metric (how the interactions are measured) is key to the interpretation of most results in this paper, please explain why you chose AUC instead of other metrics, and why "AUC up to early stationary phase". Has this approach been used in previous work? Would the conclusions hold with other metrics (e.g. yield), and if not why? Please clarify.
Also , Fig 1a schematic says "exponential phase" AUC while Fig 1 legend says "The area under the curve of the early stationary phase (AUC) was used as the final growth measurement." (L499), and in the Methods Line 256 it says "Growth curves were first trimmed at strain-specific time points when they reached stationary phase ". Please use consistent terminology and clarify how reaching stationary phase was defined.

3. I think the authors should tone down some of the claims about the broader ecological relevance of their findings.
For instance:
L26-L28. "This demonstrates the ecological relevance of V. parvula, a species that, despite being commonly present in human gut microbiomes, is generally found at low levels. Overall, the comprehensive dataset and mechanistic insights presented here provide a foundation for rational strategies to manipulate microbiome composition and function."
and
L50-51. "We used these data to prioritize the study of molecular mechanisms behind specific interactions, which enables establishing rational strategies to manipulate microbial community composition and function."
These statements should be rephrased to more explicitly and realistically acknowledge the limitations of the study (indirect, pairwise, spent media assay) and to frame the broader implications of the mechanisms observed here as a starting point for future testing.

4. Some of the results are idiosyncratic or largely qualitative, and there are a few places where a more quantitative analysis is warranted to support the claims made. See below for specifics.

Minor comments:

5. Fig 1A. I find the schematic not very clear. I would suggest adding more labels and arrows so it is clear there are 3 separate growth measurement conditions (rich, spent, and replenished spent medium). Also, in the AUC plot the authors show dynamics where AUC follows the following order AUC(spent medium) < AUC(replenished spent medium) < AUC(rich

medium). While this is a schematic, it could be interpreted as the null expectation. Please consider clarifying in the legend.

6. Line 97-99. “The proteomic profile revealed a decreased abundance of proteins involved in de novo nucleotide synthesis, and a concomitant increase in proteins associated with nucleotide salvage pathways (Figure 2b; Table S3).” This statement is not clear from Figure 2b. Please clarify.

7. Figure 2c and 2d. Please add statistics so one can assess the significance of the effects.

8. Figure 2c. Why include “Ribose”? More generally, what is the rational for including a sugar in this assay? What hypothesis is it testing? Please clarify.

9. L108. “Using dynamic light scattering, we observed particles with an average diameter of 71 nm in C. perfringens spent medium, while rich medium contained particles with an average diameter of 149 nm (Figure S1). “ Are these the same type of particles but different sizes or different types of particles? Please clarify.

10. Fig 1d. In addition to degrading the vesicles, can boiling the spent media affect other compounds/ molecules that may be heat sensitive?

11. L517 (Fig 3 legend label). “Negative growth interactions are largely explained by lower pH” seems too strong of a statement here given that it is shown for a subset (4 Bacteroidota) of the strains.

12. L153. “The results showed that V. parvula increased the pH of the medium, with the strongest activity observed at pH 5 ”. What does “the strongest activity” mean? By how much? Please quantify the magnitude of change.

13. L155. “This pH-increasing effect gradually diminished as the initial pH increased from 5 to 7, indicating that its ability to raise pH is most pronounced under acidic stress.” Could the diminished effect at higher initial pH simply reflect less scope for increase (ie starting at pH 7 leaves much less room to increase than starting at pH 5)?

14. L160. “Similarly, when V. parvula was grown in the spent medium of S. parasanguinis, the pH increased modestly to 6.2, and P. merdae growth was improved” Is the effect significant? Please add statistics to Fig 4b.

15. Why does P. merdae seem to grow better at pH 6.2 than pH 7 (Fig 4b) since they are pH-sensitive?

16. Figure 4d. Can V. parvula use arginine or guanine for growth? If so how much growth is due to growth in rich medium versus growth in arginine or guanine?

17. L229: “Our study also recapitulated the interactions of Veillonella parvula with lactate-producing species, and its capacity to increase environmental pH.” This reads as an overstatement given what is shown in this study, so please rephrase.

18. L258-261. The sentence is unclear. Please rephrase.

(Remarks on code availability)

Version 1:

Reviewer comments:

Reviewer #1

(Remarks to the Author)
I would like to thank the authors for their additional efforts in addressing the issues raised.

1a. The results presented in the new Supplementary Figure 1 are very informative. When compared directly with Figure 1b, they demonstrate which interactions disappear and which remain when nutrients and pH are replenished and adjusted, respectively. In my opinion, the interactions that remain are the most interesting findings of the dataset.
This information should be presented alongside Figure 1b in the main Figure 1.

1b. The inhibition of the specialised mucus degrader A. muciniphila by multiple Bacteroides/Phocaeicola species that are known to occupy a similar niche (mucus layer) in vivo is puzzling. I believe a discussion around these results is needed.

(Remarks on code availability)

Reviewer #2

(Remarks to the Author)
The authors have addressed my comments. The revised manuscript is much improved, and I have no further comments.

(Remarks on code availability)

Open Access This Peer Review File is licensed under a Creative Commons Attribution 4.0 International License, which permits use, sharing, adaptation, distribution and reproduction in any medium or format, as long as you give appropriate credit to the original author(s) and the source, provide a link to the Creative Commons license, and indicate if changes were made.
In cases where reviewers are anonymous, credit should be given to 'Anonymous Referee' and the source.
The images or other third party material in this Peer Review File are included in the article's Creative Commons license, unless indicated otherwise in a credit line to the material. If material is not included in the article's Creative Commons license and your intended use is not permitted by statutory regulation or exceeds the permitted use, you will need to obtain permission directly from the copyright holder.
To view a copy of this license, visit https://creativecommons.org/licenses/by/4.0/

We thank the reviewers for their insightful comments that we feel greatly improved our manuscript. We have now addressed all the points raised by the reviewers. The major changes that we have made are:

- Confirmed that the results of static OD measurements correlate with an agitated culture.
- Clarified why we use AUC as a metric and showed that the results would be similar if final yield was used.
- Characterized the membrane vesicles and rich medium by electron microscopy.
- Characterized the growth of every strain in a range of pH values to show that pH seems to be a major driver of growth inhibition in our setup.
- Expanded the number of strains profiled in spent medium adjusted to pH of rich medium.
- Performed metabolomics experiments to quantify ammonia, amino acids and nucleic acid components produced by *V. parvula*.
- Included quantification and statistical analyses for multiple experiments.

Below we address all of the comments point-by-point.

Reviewer #1 (Remarks to the Author):

The manuscript by Buyanbadrakh et al. investigates pairwise interactions among 36 representative gut bacterial strains, reporting that approximately 25% of interactions result in growth inhibition and 4% lead to growth promotion. The authors also provide additional insights into specific interactions, particularly those between M. gnavus and C. perfringens, and between Veillonella and P. merdae. Overall, the manuscript is clearly written, the data is well presented, and the figures are of high quality. The methods are mostly sound, and the selection of representative strains is reasonably justified. The incorporation of proteomic analyses of selected bacteria grown in spent versus fresh media is a strength.

However, I remain uncertain about the ecological significance of the findings, and the extent to which they advance our understanding of these interactions is somewhat limited, as I outline in my comments below. Despite these limitations, the study provides a systematic dataset of interactions between representative gut bacteria. This offers a useful foundation for future efforts to analyse microbe-microbe interactions.

1. Many of the reported interactions appear to be driven largely by acidification of spent media. It is unclear how relevant these pH shifts are for bacterial interactions in vivo, given that distal gut pH is generally stable and any local drops are expected to be transient? Therefore, I would have expected that the interactions/growth in spent media were tested after adjusting the pH of spent media. Indirectly adjusting pH by diluting with rich media, as the authors did, does not allow to disentangle the effect of cross-feeding/nutrient depletion/inhibitory metabolites vs. pH, as it also dilutes spent media. Unless the authors can convincingly discuss the ecological relevance of such changes in pH, further experiments on how pH really affects results would be important, i.e. testing interactions with spent media with pH adjusted only (no/minimal dilution).

We agree with the reviewer that a large fraction of the negative growth interactions observed in our setup might not be relevant in the context of a human gut, and this was exactly the point we wanted to raise awareness with Figure 3. We now provide a framework to distinguish pH-driven effects from other effects.

We first characterized the growth of all species in rich medium adjusted to a range of pH values between 5 and 7. This showed that for most strains, growth at different pH values relative to pH 7, mirrored the effects seen in spent media with similar pH. We include these as a new Supplementary Fig. 3.

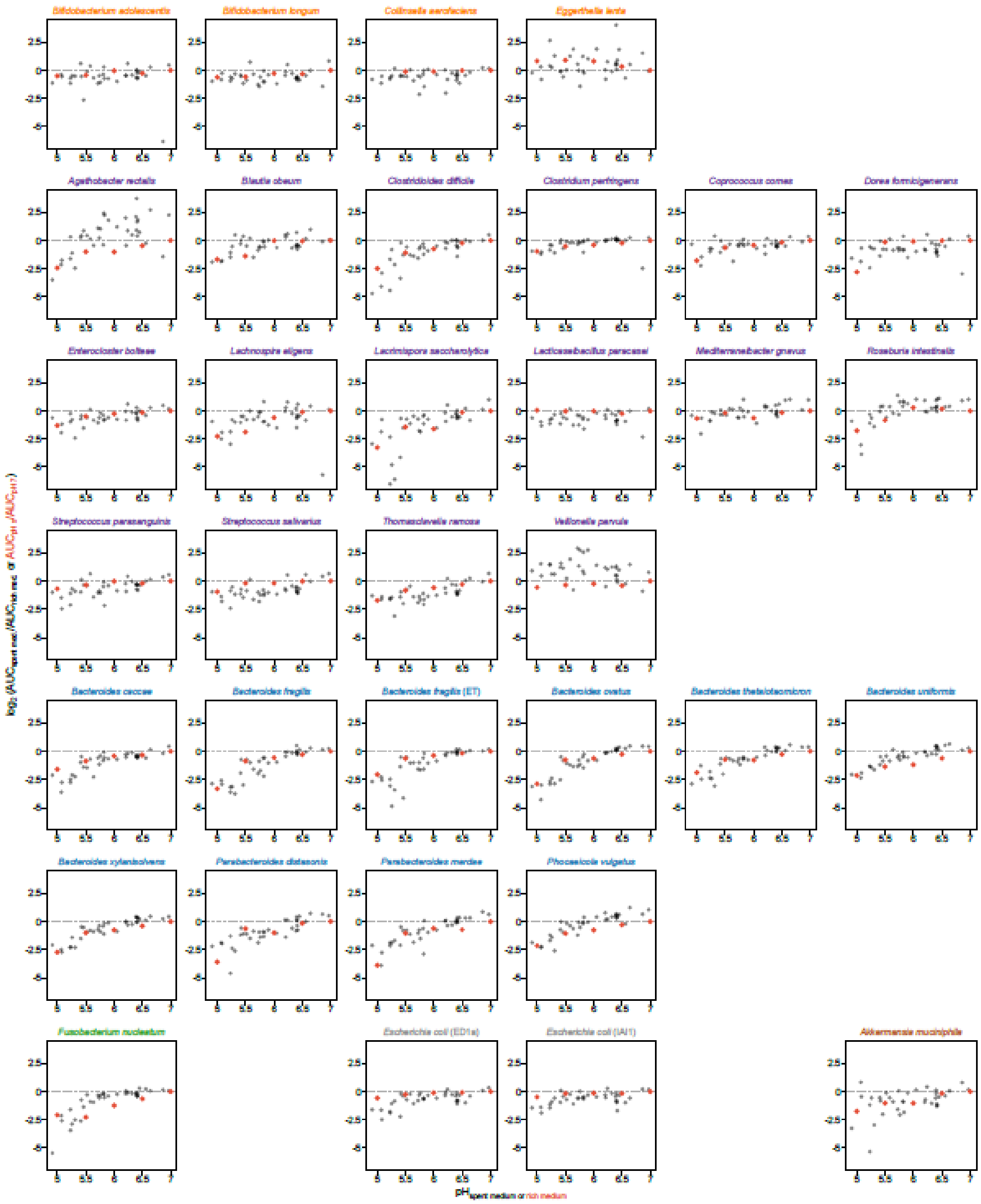

We then used these results to estimate the growth effects of each strain at the pH of each spent medium, which showed a reasonable correlation with the measured growth effects ($r = 0.54$, $p = 10^{-95}$). We include these results as a new panel b in Figure 3.

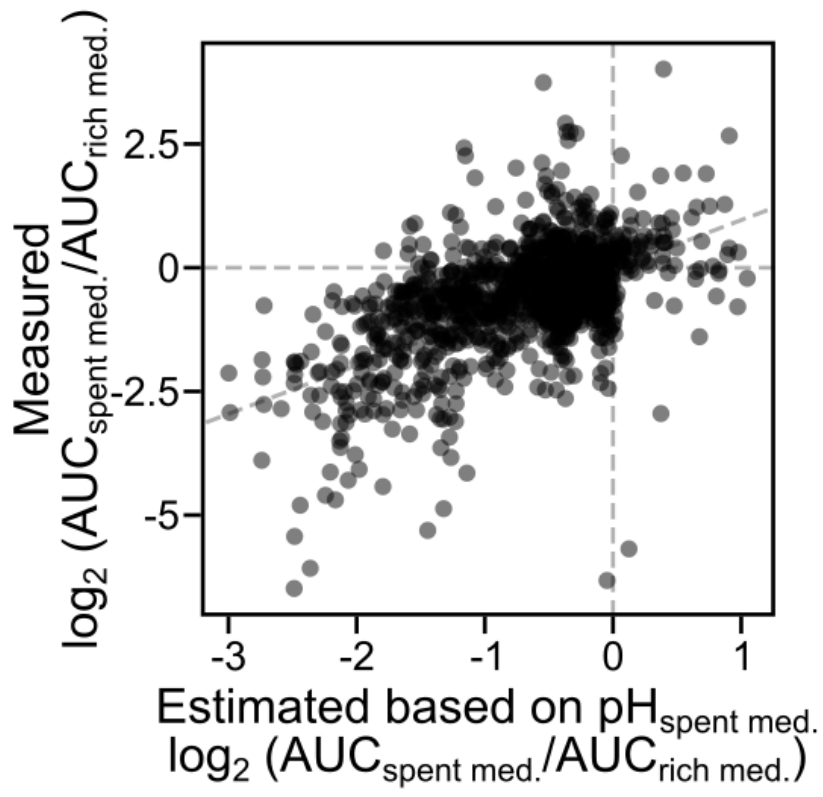

To directly assess specific interactions, we performed the experiment suggested by the reviewer (adjusting the spent medium pH) in a selected group of 56 species pairs. This limited set was enriched in negative interactions, and confirmed that after pH adjustment most interactions became neutral. We include these results as a new panel c in Figure 3.

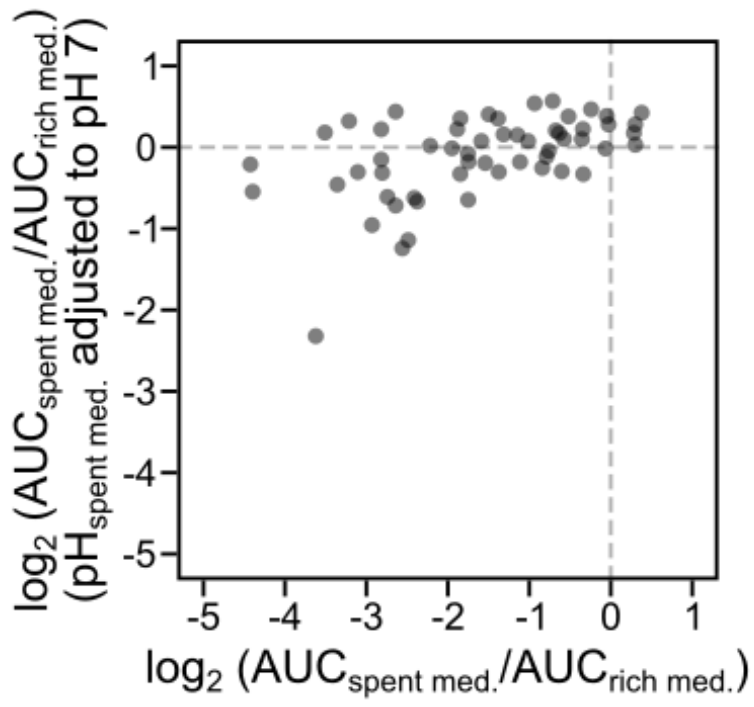

Still, we observed some interactions that remained inhibitory after pH adjustment, e.g., *B. fragilis* (ET) in *B. fragilis* spent medium, or *A. muciniphila* in *B. uniformis* spent medium. Interestingly, in both cases the interaction remained inhibitory in replenished medium, suggesting that there is a secreted factor from *B. fragilis* and *B. uniformis* that inhibits the growth of *B. fragilis* (ET) and *A. muciniphila*, respectively.

Action taken: Profiled the growth of all strains in rich medium adjusted to different initial pH values (new Supplementary Fig. 3), and used these results to estimate the expected results in each spent medium (new Figure 3b). Selected 56 strain pairs for further testing and shown that most interactions become neutral upon pH neutralization (new Figure 3c). Rewritten the results and discussion to include these findings.

1a. Lines 68-70: would be good to see the heatmap equivalent to Fig.1b when the spent medium was replenished with nutrients. For instance, do interactions between M. gnavus and perfringens still hold?

We have now added a new Supplementary Fig. 1 to include this.
To clarify, the *M. gnavus* and *C. perfringens* interaction is still present in replenished medium.

Action taken: We have added a new Supplementary Fig. 1.

2. The OD measurements were performed using a Cerillo Stratus plate reader, which does not incorporate shaking. This raises concerns about the accuracy of OD readings, especially for strains that tend to aggregate or form biofilms. Without agitation, cells can rapidly settle, leading to inconsistent or misleading OD values.

The authors present in Fig.1c the replicate variability (I commend the authors for reporting this), but does one know whether the device reliably captured cells in suspension rather than reading mostly settled cultures?

This is a valid concern. Our initial rationale was that cultures would not need aeration, and for most strains we did not observe any visual aggregation at the end of the experiments. Regardless, we have now performed a growth experiment with all the strains using two plate readers in parallel: one static and one on top of a shaker. The results were highly correlated (r=0.97), and thus we believe that our results under static conditions mostly reflect changes in growth.

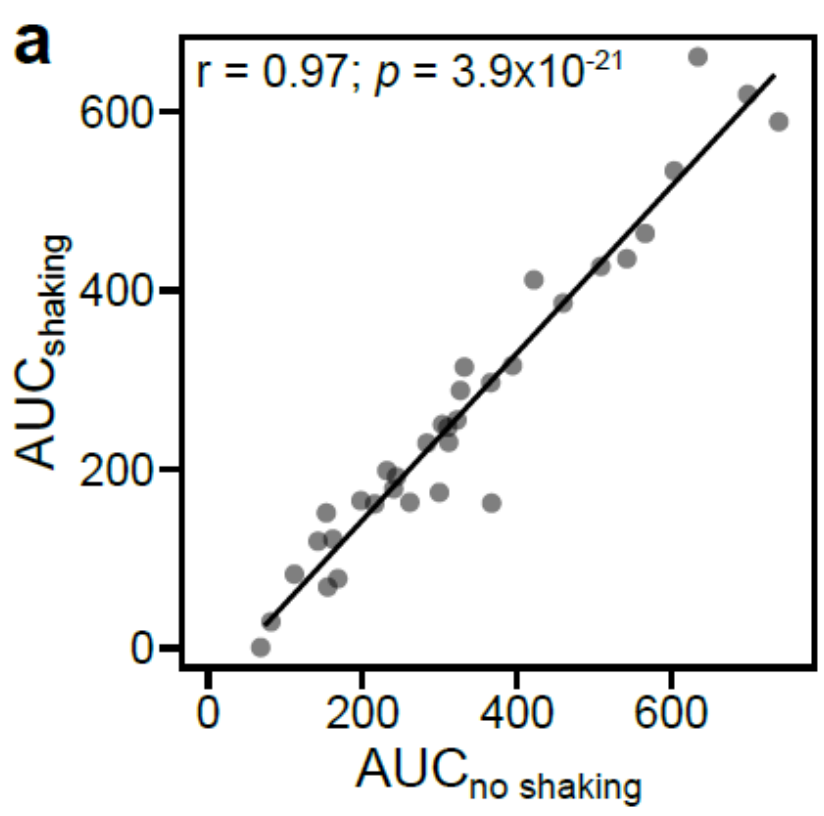

Action taken: Included a new Supplementary Fig. 5a with the results showing high correlation between static and shaking growth conditions.

3. The authors nicely show that vesicles produced by C. perfringens boost M. gnavus growth, and literature supports the presence of nucleic acids in these vesicles - but addition of nucleic acids does not boost M. gnavus growth - why? How do authors envision that vesicles containing DNA would?

We speculate that vesicles would be necessary for the uptake of nucleic acids (or other nutrients) by *M. gnavus*. Such delivery mechanisms have been described for other species (see for example review by Toyofuku et al., PMID: 36932221), and *M. gnavus* has been shown to exchange DNA with other members of the same species via vesicles (Klieve et al., PMID: 16085810). These uptake mechanisms generally involve specific receptors on the cell surface. We attempted to break any interaction by "surface shaving" the *C. perfringens* vesicles with a protease (trypsin or proteinase K). Unfortunately, we could not identify a condition (time and protease concentration) in which the vesicles remained structurally intact, but their surface proteins had been digested, which prevented us from studying this further. For this reason, we opted to be careful in our conclusions of these results.

Action taken: We have rephrased the conclusion sentence of this section: "In summary, *C. perfringens* promotes the growth of *M. gnavus* by releasing extracellular vesicles, which might facilitate the uptake of growth enhancing components, such as nucleic acid components present in these vesicles.".

4. For the proteomics analysis, bacterial cells were harvested after 24 h of growth (methods). Is 24 hours an appropriate time point to capture biologically relevant differences in the proteome? Table S1 suggests cultures would be already in the stationary phase at 24h, for both M. gnavus and V. parvula.

We reasoned that, since in both cases we observe a difference in final yield, the proteome in the stationary phase would reflect the status of the cell that led to the growth changes. We have now repeated both proteome measurements in the exponential and stationary phases, and observed a reasonable correlation between the two measurements (for *R. gnavus*, r = 0.36; for *V. parvula*, r = 0.60). log2_fc corresponds to the log2 fold-change between spent medium and rich medium, as in the volcano plots.

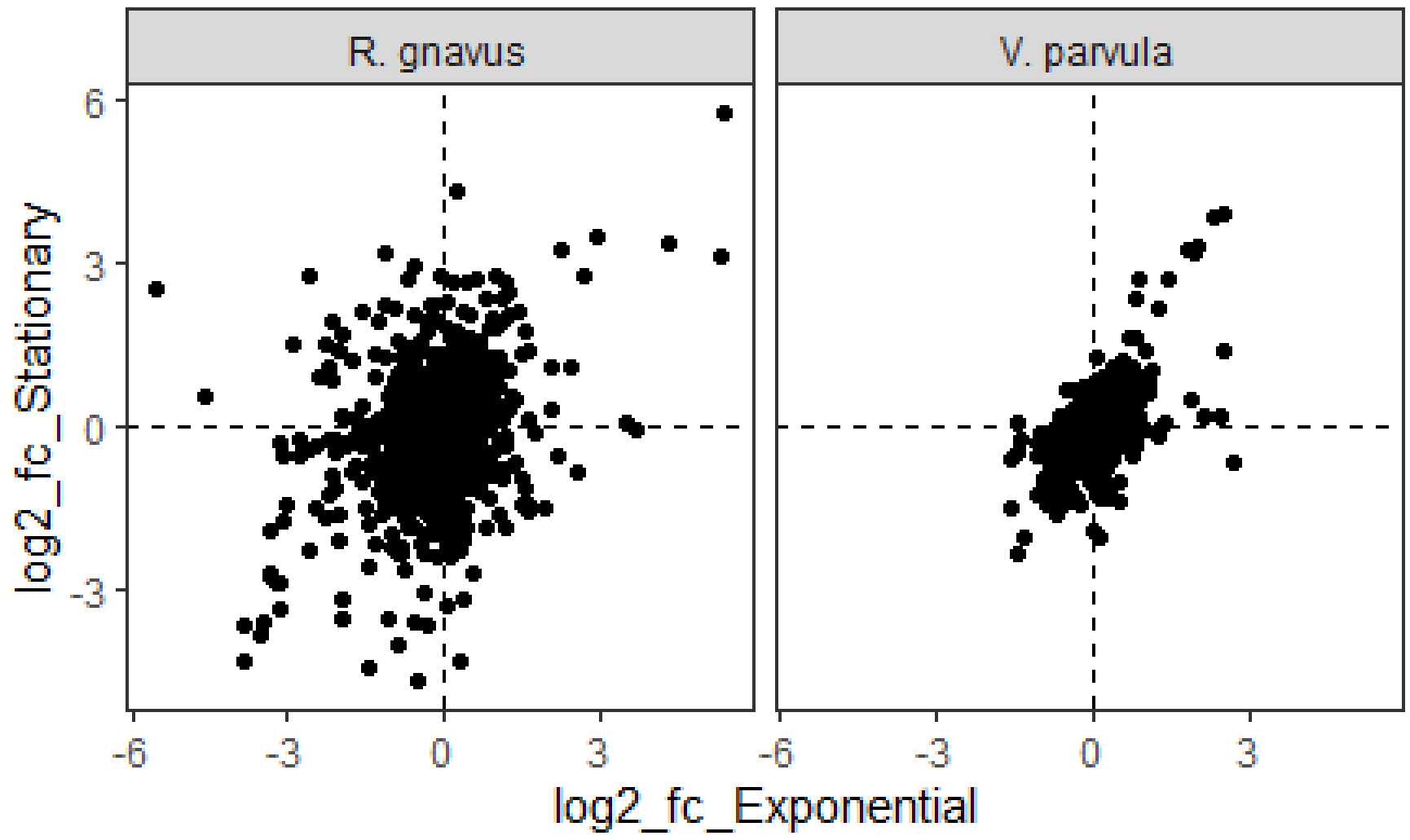

It should be noted that this is a level of correlation that is expected between two independent proteomics experiments. We compared the new measurements in stationary phase to the ones included in the manuscript and observed similar levels of correlation (for *R. gnavus*, r = 0.53; for *V. parvula*, r = 0.40).

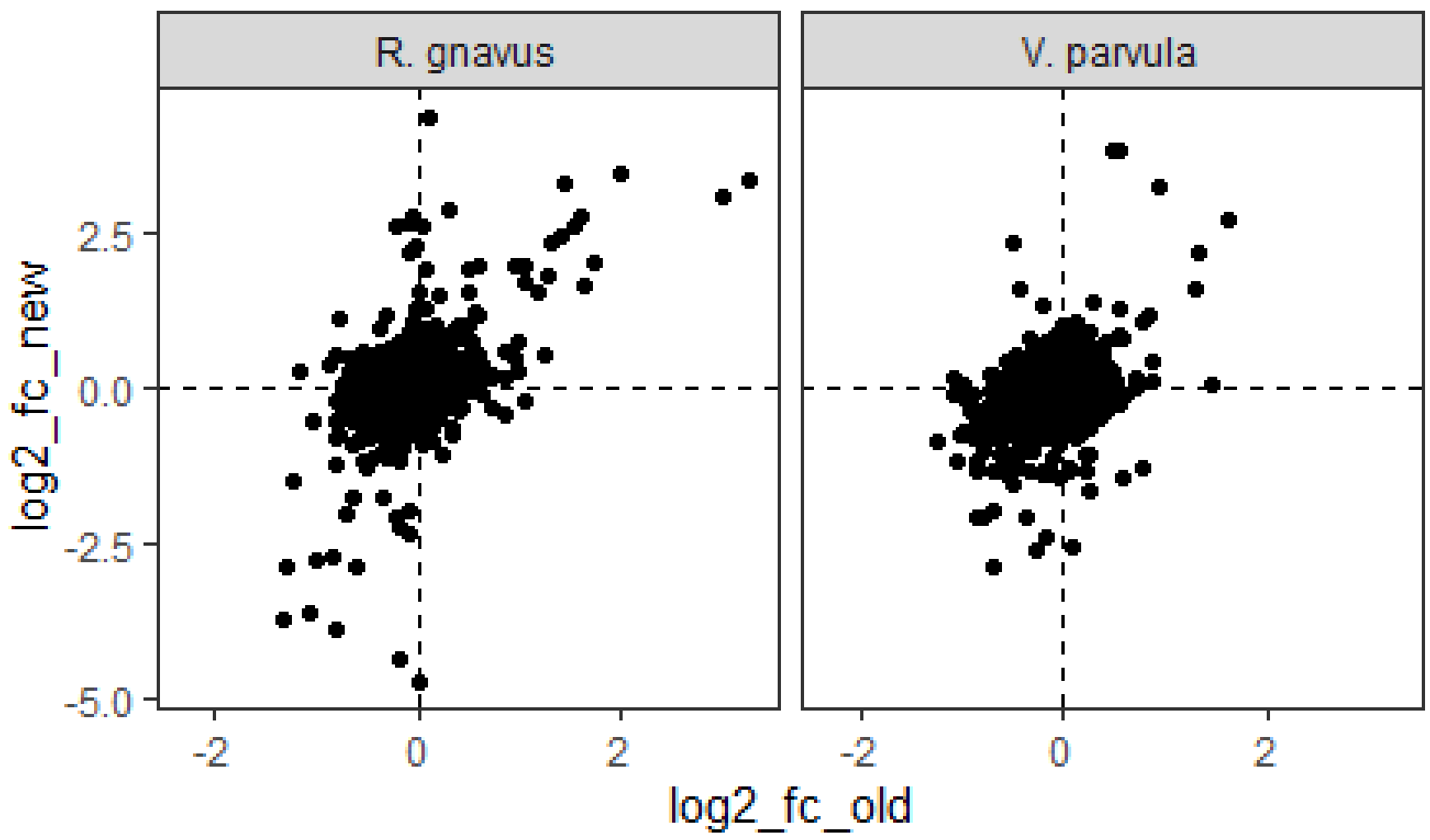

Action taken: We have clarified in the methods section why we performed experiments at 24 h: "Bacterial cells were harvested by centrifugation at 3000 × g for 15 min at 4 °C from a 24 h culture.

This timepoint was chosen since we observed a change in final OD_{600}, and expected that molecular changes would reflect the status of the cell that led to the growth changes."

5. The mechanism via which Veilonella is increasing pH should be further explored: ammonia production would be an obvious one, as levels of the enzyme guanidine deaminase are increased (Fig.4c), and this is known to catalyse the hydrolytic deamination of guanine to xanthine and ammonia. Has ammonia production been measured?

We have now measured the levels of ammonia, amino acids, and nucleic acid components in the spent medium of *V. parvula* grown in *S. parasanguinis* spent medium compared to rich medium. While ammonia levels were not altered, we observed changes in purine metabolism (increase of guanine, guanosine, adenine, hypoxanthine, and inosine, and decrease of adenosine) and in arginine metabolism (decrease of arginine, and increase of citrulline, ornithine, aspartic acid, and glutamine). This suggests that the observed proteome changes reflect limited availability of some of these metabolites. Thus, the effect of guanine on the pH might reflect an increase in growth of *V. parvula* (that we noticed based on reviewer #2 point 16). Since *V. parvula* can ferment organic acids for energy (e.g., lactate produced by *Streptococci*), an increase in growth would reduce the concentration of organic acids and potentially increase the pH.

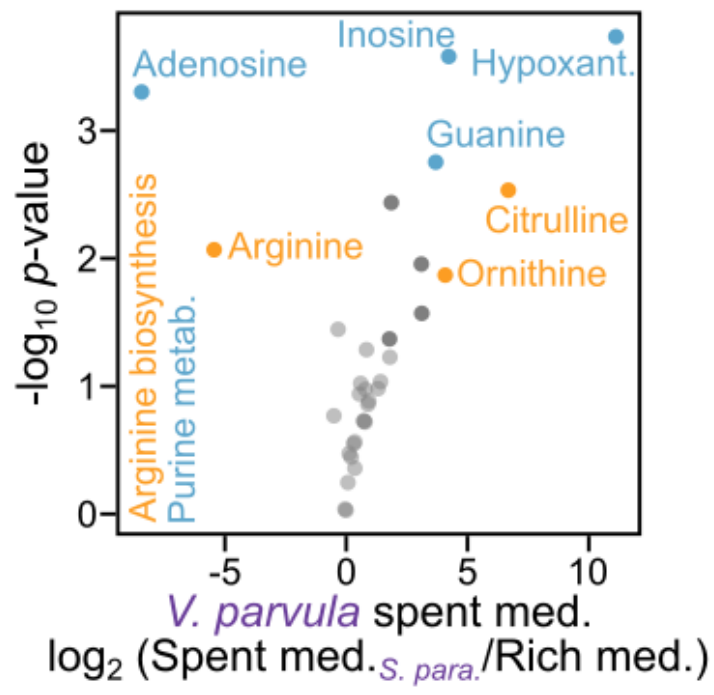

Action taken: We performed a metabolomics analysis and re-interpreted our observations based on the results.

6. The discussion lacks depth in addressing how the results from these in vitro pairwise assays can be generalized to community level dynamics under the physiological conditions of the gut. A broader consideration of how these findings translate to the complex, spatially structured, mostly buffered environment of the intestine is missing.

Action taken: We have expanded the discussion to highlight that it remains to be studied if spatial segregation is sufficient to create local pH environments or if they are buffered by the gut, and thus it is important to distinguish inhibitory interactions that are not driven by pH changes.

7. In addition, some statements are insufficiently explained. For instance: "M. gnavus produces an anti-C. perfringens bacteriocin emphasizing strong growth dynamics between these species." What do the authors implicate by this, I do not see any inhibition of perfringens growth in M. gnavus spent media. Is this because a different strain was used?

We wanted to highlight that even though we observed growth promotion from *C. perfringens* to *M. gnavus*, there are described interactions in the opposite direction. The reason why we did not observe this particular interaction is that it likely requires trypsin for activation (PMID: 8215361).

Action taken: To avoid confusion, we have removed this statement.

Other points:

8. Results, lines 74-78: authors start the paragraph with "We next explored if inhibitory interactions were mostly reciprocal…". The way it is currently written, it gives the impression that all percentages reported are a % of inhibitory interactions, not of the total interactions tested. This needs to be rephrased for clarity.

Action taken: We have clarified that the percentages refer to all pairwise interactions.

Reviewer #2 (Remarks to the Author):

Buyanbadrakh et al profile the pairwise interactions between 36 gut bacterial strains using a spent medium assay. The authors report that most interactions are inhibitory and due to resource depletion or medium acidification. The authors then focus on a few positive interactions to investigate mechanisms. The dataset is valuable and the follow-up mechanistic experiments on a few selected interactions are interesting. My main comments are about clarifying how interactions are quantified, tone down a few general claims so they more closely match the experiments and data, and add quantitative values/ statistics when missing. See my comments below for details.

Main comments:

1. The authors state that "Most interactions were inhibitory and largely explained by resource depletion or medium acidification." (L19) and "Widespread growth inhibiting and growth promoting ecological interactions among human gut microbes." (Figure 1 legend; L94). However, Figure 1d does suggest that most interactions are actually neutral under the 2-fold change threshold. I think that the statement that "Most interactions were inhibitory" should be clarified and rephrased.

Action taken: We have clarified in the abstract that "Here, we systematically profiled 1,224 pairwise growth interactions among 36 representative gut bacterial strains using a spent medium assay, and observed that when two species interacted, the interactions were mostly inhibitory.", and have rephrased the Figure 1 legend title to: "Growth interaction profiling among human gut microbes."

It would also be helpful to show the following heatmaps: a heatmap like Fig 1b for the replenished medium data and heatmaps with the 2-fold change threshold for both the spent medium and the replenished medium. This would make it clearer which pairwise interactions are inhibitory and more likely driven by resource depletion vs other mechanisms of inhibition, and which interactions are neutral or positive.

Action taken: We have now added a new Supplementary Fig. 1 to include this. We have also marked both in Figure 1b and in this new figure, which interactions are above the two-fold change threshold.

2. L60. "Growth dynamics were quantified by calculating the area under the growth curve (AUC)." Because this metric (how the interactions are measured) is key to the interpretation of most results in this paper, please explain why you chose AUC instead of other metrics, and why "AUC up to early stationary phase". Has this approach been used in previous work? Would the conclusions hold with other metrics (e.g. yield), and if not why? Please clarify.

Also , Fig 1a schematic says "exponential phase" AUC while Fig 1 legend says "The area under the curve of the early stationary phase (AUC) was used as the final growth measurement." (L499), and in the Methods Line 256 it says "Growth curves were first trimmed at strain-specific time points when they reached stationary phase ". Please use consistent terminology and clarify how reaching stationary phase was defined.

Our rationale for using the AUC is that this metric captures changes in both yield and growth rate in a non-parametric fashion, i.e., without requiring modeling of the growth curve. The reason for using the AUC only until early stationary phase (with a species-specific cutoff) was to account for different species having different growth rates. Using a later time point for all species would lead to species that

have fast growth rates to show smaller fold-changes, since the AUC would be dominated by stationary phase. Further, for some species, we observed a decrease in OD after some time in stationary phase. The figure below shows the average OD at each time point for each species in the rich medium condition, as well as the time cutoff (i.e., data to the right of the dashed line was not used for the AUC calculation).

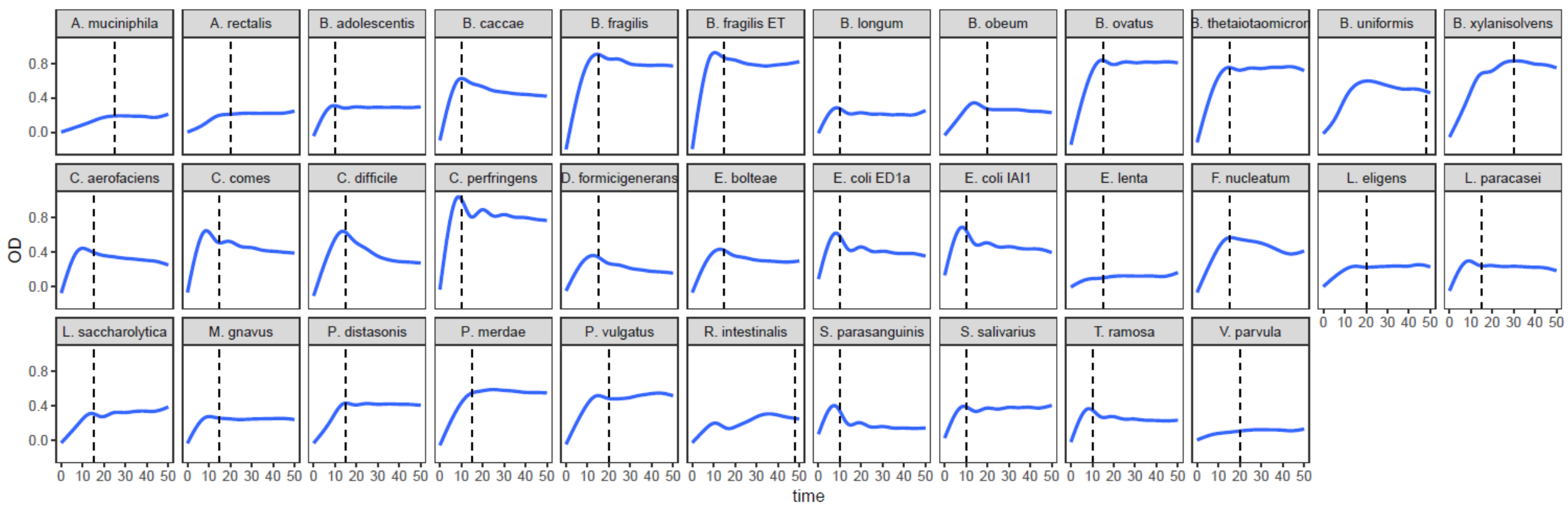

We have repeated the analysis using yield by calculating the average OD within a 30-minute window around the cutoff time point above. The calculated $\log_2$ fold-changes (spent medium or replenished medium vs rich medium) were strongly correlated with those calculated with AUC (r=0.92).

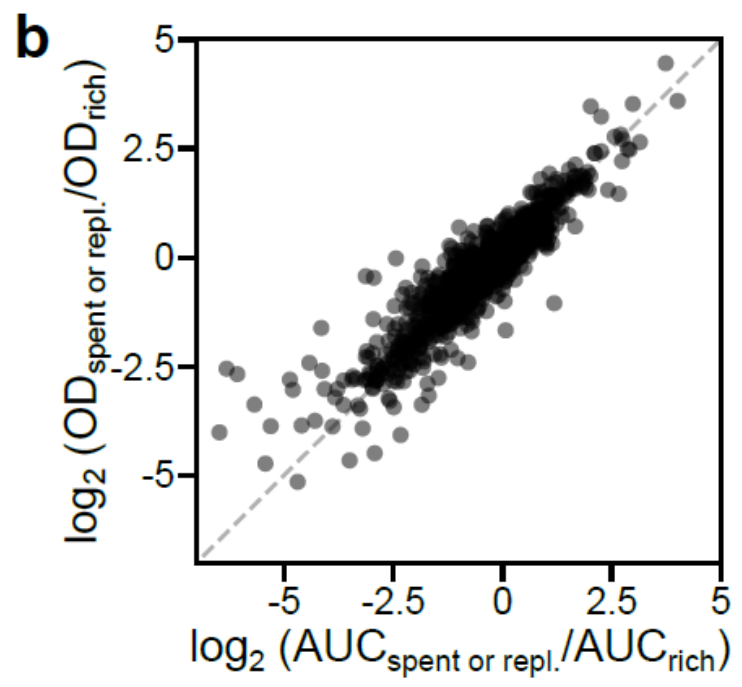

While there are some specific conditions that differ, we believe that using one or the other metric will always have advantages or disadvantages. Since we provide all the raw growth curve data (https://zenodo.org/records/17456178), future studies can carefully evaluate the reason for changes in the AUC. We now include these results as Supplementary Fig. 4b and clarify in the methods section why we chose AUC as the metric: "Performing the same analysis using the average OD_{600} within a 30 min window around the trimming time point resulted in similar results to using the AUC (Supplementary Fig. 5b; $r = 0.92$, $p < 10^{-16}$). We opted for AUC to reflect both changes in yield and growth rate."

We apologize for having inconsistently named the time cutoff, and we have now revised the text in all places to "early stationary phase".

<u>Action taken:</u> We have clarified in the discussion that growth curves can be visually inspected or fitted to extract growth parameters to evaluate the reason for changes in the AUC. We have included the analysis with OD instead of AUC as a new Supplementary Fig. 5b, and have explained our rationale for choosing AUC in the methods section. We have also consistently named the cutoff as "early stationary phase".

3. I think the authors should tone down some of the claims about the broader ecological relevance of their findings.

For instance:

L26-L28. "This demonstrates the ecological relevance of V. parvula, a species that, despite being commonly present in human gut microbiomes, is generally found at low levels. Overall, the comprehensive dataset and mechanistic insights presented here provide a foundation for rational strategies to manipulate microbiome composition and function."

and

L50-51. "We used these data to prioritize the study of molecular mechanisms behind specific interactions, which enables establishing rational strategies to manipulate microbial community composition and function."

These statements should be rephrased to more explicitly and realistically acknowledge the limitations of the study (indirect, pairwise, spent media assay) and to frame the broader implications of the mechanisms observed here as a starting point for future testing.

Action point: We have toned down both statements:

- Abstract: "Although *V. parvula* is commonly present in human gut microbiomes, it is generally found at low levels. Given the spatial organization of bacteria in the gut, the local pH modulation by *V. parvula* might support the growth of acid-sensitive strains. Overall, the comprehensive dataset and mechanistic insights presented here provide a starting point to understand microbiome composition and function."
- Introduction: removed "which enables establishing rational strategies to manipulate microbial community composition and function"

4. Some of the results are idiosyncratic or largely qualitative, and there are a few places where a more quantitative analysis is warranted to support the claims made. See below for specifics.

We have now added quantification or statistical tests where requested (see below). All raw data and quantification are also provided in the supplementary tables.

Minor comments:

5. Fig 1A. I find the schematic not very clear. I would suggest adding more labels and arrows so it is clear there are 3 separate growth measurement conditions (rich, spent, and replenished spent medium). Also, in the AUC plot the authors show dynamics where AUC follows the following order AUC(spent medium) < AUC(replenished spent medium) < AUC(rich medium). While this is a schematic, it could be interpreted as the null expectation. Please consider clarifying in the legend.

Action taken: We have followed the reviewer suggestions by changing the figure and the legend to "The area under the curve of the early stationary phase (AUC) for each of the conditions was used as the final growth measurement (the order of the schematic growth curves is used for illustration purposes only).".

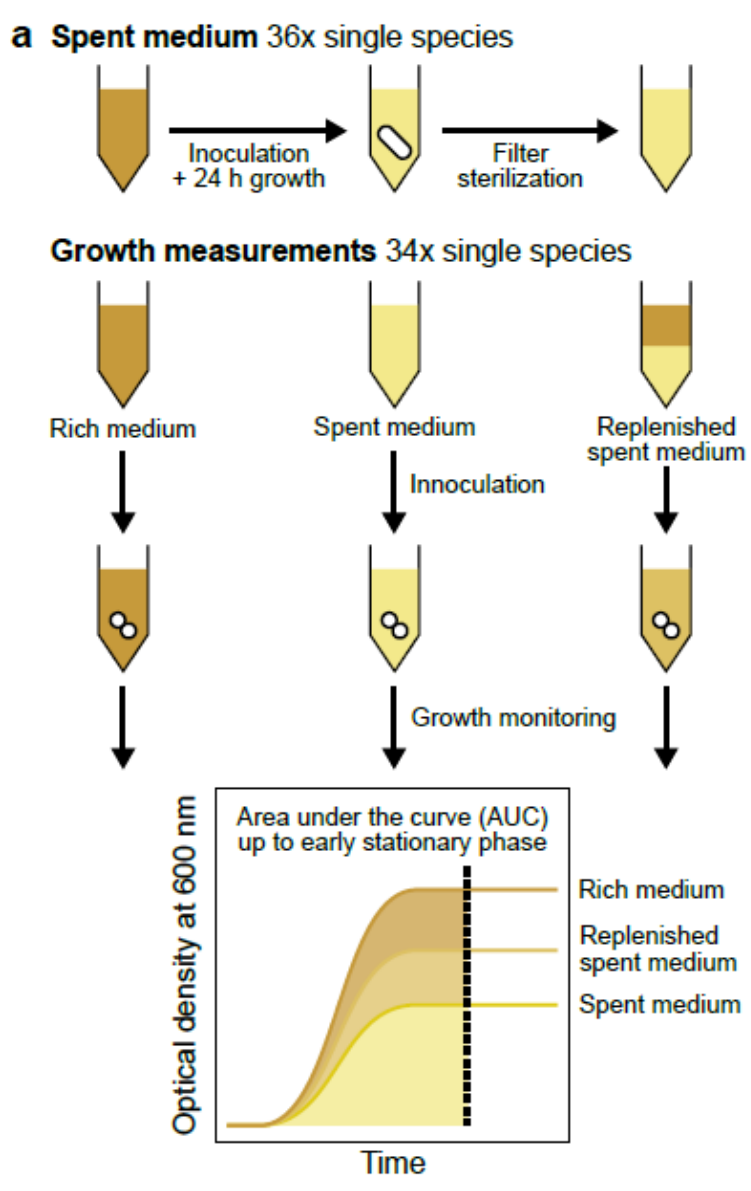

6. Line 97-99. "The proteomic profile revealed a decreased abundance of proteins involved in de novo nucleotide synthesis, and a concomitant increase in proteins associated with nucleotide salvage pathways (Figure 2b; Table S3)." This statement is not clear from Figure 2b. Please clarify.

Action taken: We have now redone Figure 2b by separating which proteins are in involved in nucleotide synthesis vs metabolism or degradation according to KEGG.

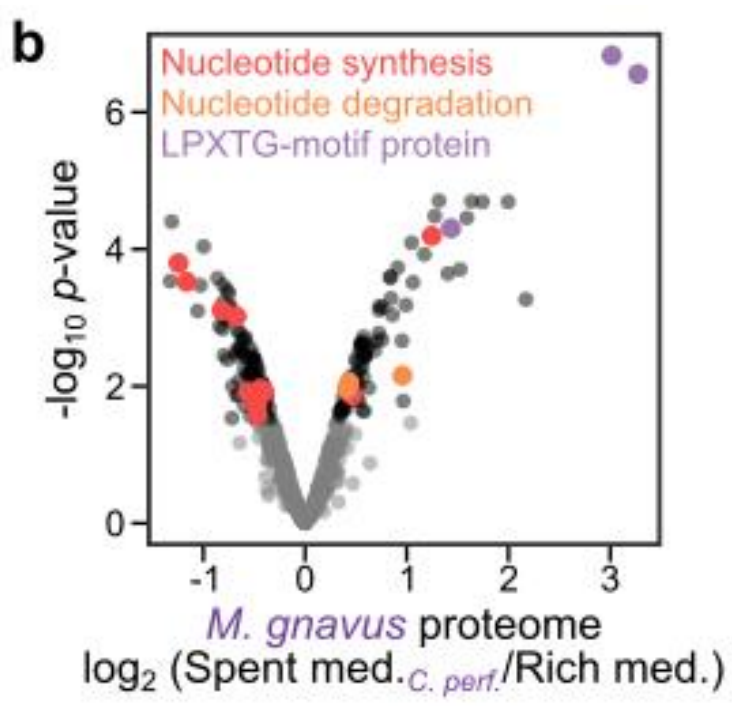

7. Figure 2c and 2d. Please add statistics so one can assess the significance of the effects.

Action taken: We have now added statistical tests to the results section and in Supplementary Table 4 and 5.

8. Figure 2c. Why include "Ribose"? More generally, what is the rational for including a sugar in this assay? What hypothesis is it testing? Please clarify.

We included ribose to distinguish if *M. gnavus* was utilizing the sugar component of nucleosides (or the nucleobase), in case we had observed an effect with nucleosides.

Action taken: We have clarified in the results that ribose was used as a way to test the sugar component of nucleosides: "Thus, we tested whether nucleic acid components could promote the growth of M.

gnavus by culturing it in media supplemented with nucleobases, nucleosides, the sugar component (ribose), and nucleic acids."

9. L108. "Using dynamic light scattering, we observed particles with an average diameter of 71 nm in C. perfringens spent medium, while rich medium contained particles with an average diameter of 149 nm (Figure S1). " Are these the same type of particles but different sizes or different types of particles? Please clarify.

We have now performed negative stain electron microscopy of the particles that are present in rich medium and of the *C. perfringens* vesicle fraction. The particles are visibly different in their morphology, and thus we believe that the *C. perfringens* fraction is not merely smaller particles from rich medium.

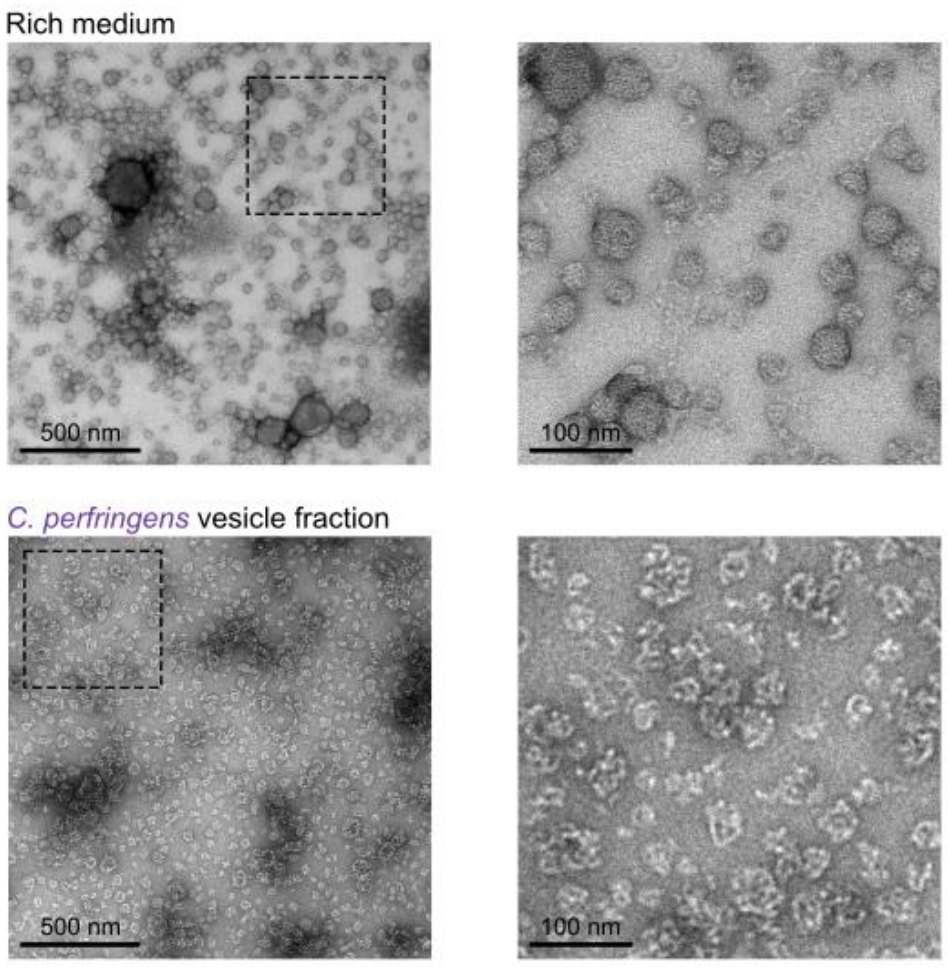

Action taken: We added these results to Supplementary Fig. 2b and to the results section: "These particles were visibly different from particles isolated from rich medium, with a hollow core reminiscent of vesicles (Supplementary Fig. 2b).".

10. Fig 1d. In addition to degrading the vesicles, can boiling the spent media affect other compounds/ molecules that may be heat sensitive?

Absolutely. The negative stain electron microscopy showed that the vesicles were visibly different after boiling, but it is possible that other components are heat sensitive.

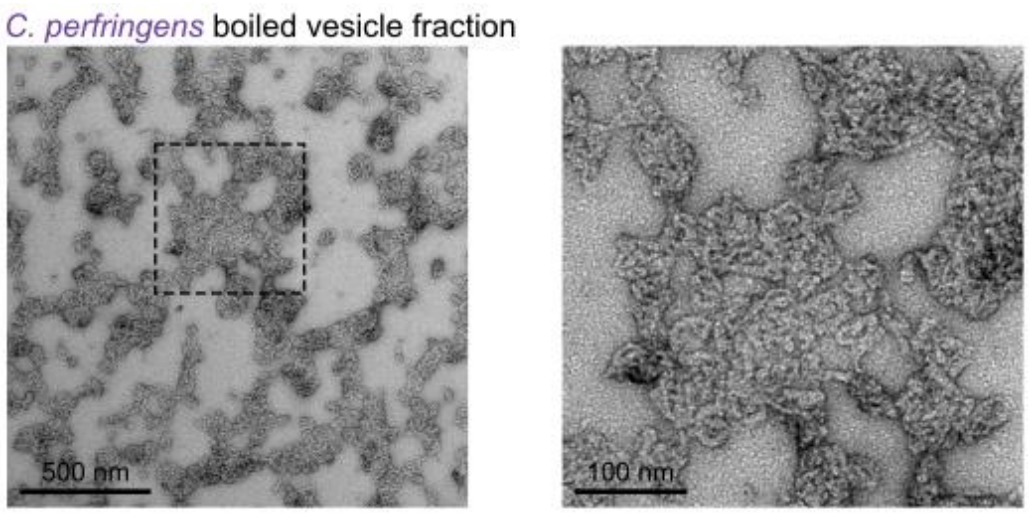

Action taken: We have clarified in the results: "Furthermore, heat treatment of the vesicle fraction abolished the growth-promoting effect ($p = 0.80$ on a one-sample t-test; Figure 2d), and led to a change in the morphology of the vesicles (Supplementary Fig. 2b). This indicated that heat either disrupted vesicle structure or that the growth-promoting components are heat-labile."

11. L517 (Fig 3 legend label). "Negative growth interactions are largely explained by lower pH" seems too strong of a statement here given that it is shown for a subset (4 Bacteroidota) of the strains.

Action taken: We have modified the figure legend to: "Negative growth interactions are correlated with lower environmental pH."

12. L153. "The results showed that V. parvula increased the pH of the medium, with the strongest activity observed at pH 5 ". What does "the strongest activity" mean? By how much? Please quantify the magnitude of change.

Action taken: We have clarified the magnitude of increase: "The results showed that *V. parvula* increased the pH of the medium, with the strongest increase observed at pH 5, in which the final pH raised by 0.85 units." The results of individual replicates are also available in Supplementary Table 9.

13. L155. "This pH-increasing effect gradually diminished as the initial pH increased from 5 to 7, indicating that its ability to raise pH is most pronounced under acidic stress." Could the diminished effect at higher initial pH simply reflect less scope for increase (ie starting at pH 7 leaves much less room to increase than starting at pH 5)?

We agree that that is most likely the case, and that *V. parvula* is attempting to neutralize the pH.

Action taken: We have rephrased the sentence to make it clearer: "This pH-increasing effect gradually diminished as the initial pH increased from 5 to 7, suggesting that under acidic stress *V. parvula* attempts to neutralize the pH."

14. L160. "Similarly, when V. parvula was grown in the spent medium of S. parasanguinis, the pH increased modestly to 6.2, and P. merdae growth was improved" Is the effect significant? Please add statistics to Fig 4b.

Initially we had only two replicates and therefore decided not to do statistical tests. We have now performed additional replicates and added statistical tests.

Action taken: We have now added statistical tests referring to these results in the results section and in Supplementary Table 10.

15. Why does P. merdae seem to grow better at pH 6.2 than pH 7 (Fig 4b) since they are pH-sensitive?

After including additional replicates, this effect is no longer observed. There is now a slight growth promotion at pH 7, but this effect is not significant, and no growth effect at pH 6.2.

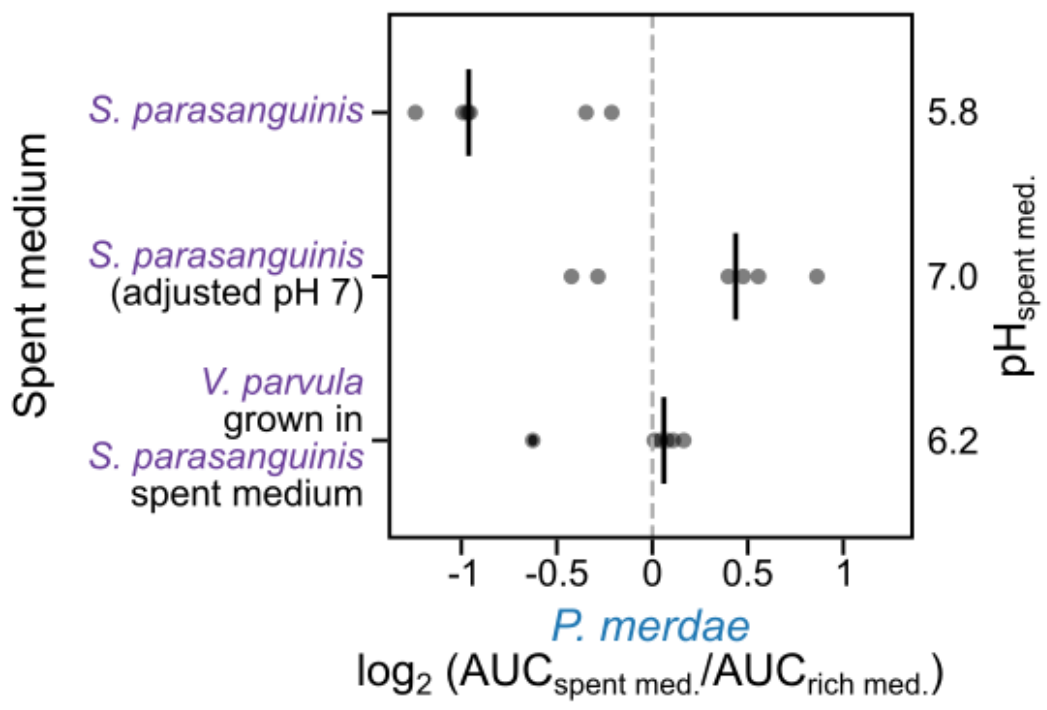

16. Figure 4d. Can V. parvula use arginine or guanine for growth? If so how much growth is due to growth in rich medium versus growth in arginine or guanine?

V. parvula does indeed show differences in growth when rich medium is supplemented with arginine or guanine. Guanine tends to lead to higher yield, while arginine tends to lead to lower yield, particularly at pH 5. These effects could be seen in the bubble size of Figure 4d, but are clearer in the growth curves shown below. This growth promotion effect might explain the pH increasing capacity of *V. parvula*, since it can ferment organic acids, such as lactate produced by *Streptococci* (see reviewer #1 point 5).

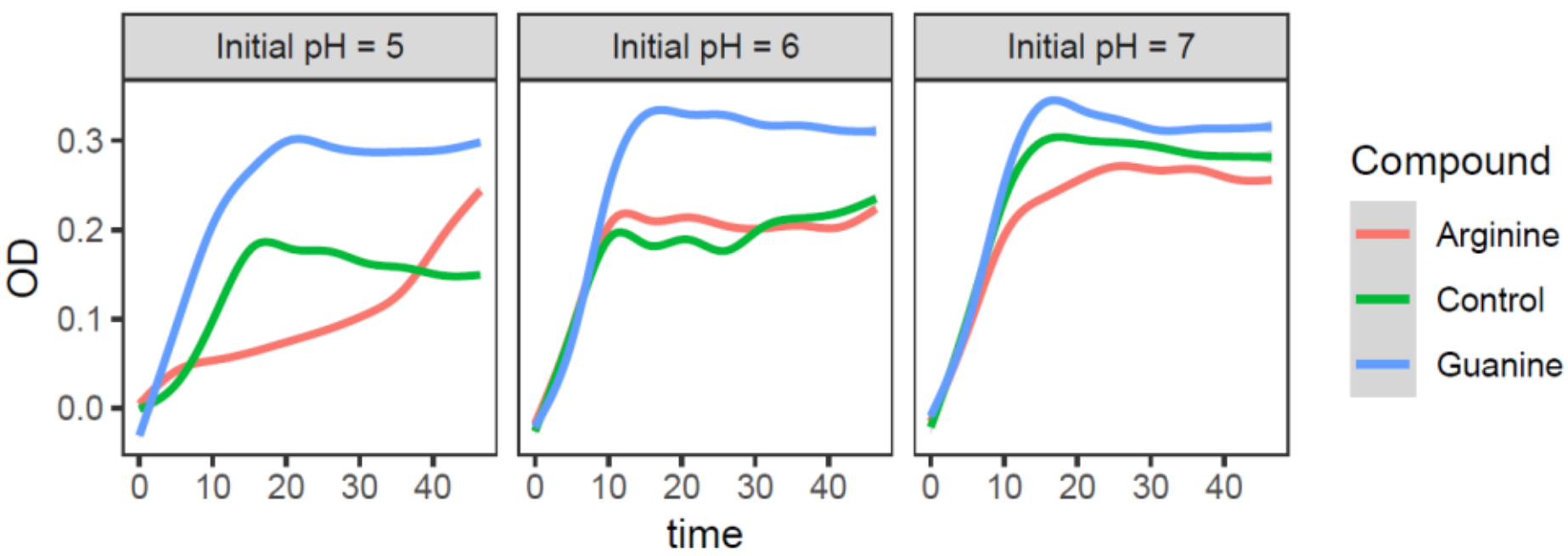

Action taken: We have made this observation clearer in the results: "Indeed, guanine supplementation increased growth of *V. parvula*, particularly at lower pH values (Figure 4d; Supplementary Table 9)."

17. L229: "Our study also recapitulated the interactions of Veillonella parvula with lactate-producing species, and its capacity to increase environmental pH." This reads as an overstatement given what is shown in this study, so please rephrase.

Action taken: We have rewritten this paragraph to reflect our findings: "Our study highlighted the capacity of *Veillonella parvula* to increase environmental pH and facilitate the growth of low pH-sensitive strains. This could explain the role in communities for this prevalent (>50% of human gut microbiomes contain this species), but low abundant species (generally <1% abundance in gut microbiomes, and 2-8% in our simple communities). The pH-increasing capacity of this species was enhanced by the addition of guanine, which also promoted its growth. Thus, the upregulation of guanine deaminase and of the arginine pathway when *V. parvula* was grown in *S. parasanguinis* spent medium could pinpoint a limited access to these metabolites. In line with this, *S. parasanguinis* is known to be strongly arginolytic.".

18. L258-261. The sentence is unclear. Please rephrase.

Action taken: We have rephrased the sentence to: “Growth curves were first trimmed at strain-specific time points when they reached early stationary phase to avoid diluting growth responses. The same time point was used for all experiments of the same strain (Supplementary Table 1).”

We again thank the reviewers for their thorough work that we believe greatly improved our manuscript.

Reviewer #1 (Remarks to the Author):

I would like to thank the authors for their additional efforts in addressing the issues raised.

1a. The results presented in the new Supplementary Figure 1 are very informative. When compared directly with Figure 1b, they demonstrate which interactions disappear and which remain when nutrients and pH are replenished and adjusted, respectively. In my opinion, the interactions that remain are the most interesting findings of the dataset.

This information should be presented alongside Figure 1b in the main Figure 1.

We agree with the reviewer that this comparison is interesting, but given the size restrictions for figures in the manuscript, we cannot find a way to fit both heatmaps in the main Figure 1. We provide the data also in Table S2, which provides a way to easily compare the two conditions.

1b. The inhibition of the specialised mucus degrader A. muciniphila by multiple Bacteroides/Phocaeicola species that are known to occupy a similar niche (mucus layer) in vivo is puzzling. I believe a discussion around these results is needed.

We thank the reviewer for highlighting this. We have added a sentence to the discussion to bring it up to the reader's attention, but refrain from speculating too much about its in vivo implication: "Of note, *A. muciniphila* growth was inhibited by the spent and replenished media of other Bacteroidota species, which could warrant further study, given that these species share a spatial niche (the mucus layer)."

Reviewer #2 (Remarks to the Author):

The authors have addressed my comments. The revised manuscript is much improved, and I have no further comments.
