# Supplementary material for: Systematic profiling of growth interactions in human gut microbiome species
Source: Nat Commun. 2026 Aug 7;17:8012. doi: 10.1038/s41467-026-76526-z (PMC13451373; doi:10.1038/s41467-026-76526-z)
Supplement: Supplementary file 4 [file 41467_2026_76526_MOESM2_ESM.pdf]

# Supplementary data

**Supplementary Data 1. Strains included in this study and pH of spent medium after 24 h growth.**

**Supplementary Data 2. Species-species growth results.**

**Supplementary Data 3. Proteome analysis of *Mediterraneibacter gnavus* grown on *Clostridium perfringens* spent medium.**

**Supplementary Data 4. Effect of *Clostridium perfringens* spent medium, nucleobases, ribose, nucleosides, and nucleic acids on the growth of *Mediterraneibacter gnavus*.**

**Supplementary Data 5. Effect of *Clostridium perfringens* spent medium and purified membrane vesicles on the growth of *Mediterraneibacter gnavus*.**

**Supplementary Data 6. Estimated effect of spent medium pH on growth of all strains.**

**Supplementary Data 7. Effect of rich medium pH on the growth of all strains.**

**Supplementary Data 8. Effect of spent medium pH on the growth of selected species combinations.**

**Supplementary Data 9. Effect of pH and addition of guanine and arginine on the growth of *Veillonella parvula* and *Parabacteroides merdae*.**

**Supplementary Data 10. Effect of *Streptococcus parasanguinis* spent medium pH, and a spent medium pH modifying strain (*Veillonella parvula*) on the growth of *Parabacteroides merdae*.**

**Supplementary Data 11. Proteome analysis of *Veillonella parvula* grown on *Streptococcus parasanguinis* spent medium.**

**Supplementary Data 12. Metabolome analysis of *Veillonella parvula* spent medium.**

**Supplementary Data 13. Effect of *Veillonella parvula* on bacterial communities.**

**Supplementary Data 14. Effect of shaking on growth measurements.**

**Supplementary Data 15. Mass spectrometry methods for the quantification of metabolites.**

**Supplementary Data 16. Primers used for qPCR.**

# Supplementary information

Supplementary fig 1-5.
